# A community approach of pathogens and their arthropod vectors (ticks and fleas) in dogs of African Sub-Sahara

**DOI:** 10.1186/s13071-021-05014-8

**Published:** 2021-11-16

**Authors:** Dieter Heylen, Michael Day, Bettina Schunack, Josephus Fourie, Michel Labuschange, Sherry Johnson, Samuel Maina Githigia, Foluke Adedayo Akande, Jahashi Saidi Nzalawahe, Dickson Stuart Tayebwa, Ortwin Aschenborn, Mary Marcondes, Maxime Madder

**Affiliations:** 1 Eco-Epidemiology Group, Department of Biomedical Sciences, Institute of Tropical Medicine, Antwerp, Belgium; 2 Interuniversity Institute for Biostatistics and Statistical Bioinformatics, Hasselt University, Diepenbeek, Belgium; 3 Department of Ecology and Evolutionary Biology, Princeton University, Princeton, NJ USA; 4 School of Veterinary and Life Sciences, Murdoch University, Murdoch, WA Australia; 5 Bayer Animal Health, Elanco Animal Health Inc., Leverkusen, Germany; 6 Clinvet LLC, Waverly, USA; 7 ClinVet International (Pty) Ltd., Bloemfontein, South Africa; 8 Clinomics, Bloemfontein, South Africa; 9 School of Veterinary Medicine, College of Basic and Applied Sciences (CBAS), University of Ghana, Accra, Ghana; 10 Department of Veterinary Pathology, Microbiology and Parasitology, University of Nairobi, Nairobi, Kenya; 11 Department of Veterinary Parasitology and Entomology, College of Veterinary Medicine, Federal University of Agriculture, Abeokuta, Nigeria; 12 Sokoine University of Agriculture, Morogoro, Tanzania; 13 Research Center for Tropical Diseases and Vector Control, College of Veterinary Medicine, Animal Resources and Biosecurity, Makerere University, Kampala, Uganda; 14 School of Veterinary Medicine, University of Namibia, Neudamm, Namibia, South Africa; 15 São Paulo State University, São Paulo, Brazil; 16 University of Pretoria, Pretoria, South Africa; 17 Clinglobal, Tamarin, Mauritius

**Keywords:** Dog, Sub-Sahara Africa, Ticks, Fleas, Vector-borne pathogens, *Ixodes*, *Haemaphysalis*, *Rhipicephalus*, *Amblyomma*, *Coxiella burnetii*

## Abstract

**Background:**

Arthropod-borne pathogens and their vectors are present throughout Africa. They have been well-studied in livestock of sub-Saharan Africa, but poorly in companion animals. Given the socio-economic importance of companion animals, the African Small Companion Animal Network (AFSCAN), as part of the WSAVA Foundation, initiated a standardized multi-country surveillance study.

**Methods:**

Macro-geographic variation in ectoparasite (ticks and fleas) and pathogen communities in dogs was assessed through molecular screening of approximately 100 infested dogs in each of six countries (Ghana, Kenya, Nigeria, Tanzania, Uganda and Namibia), both in rural and urban settings. The most important intrinsic and extrinsic risk factors within the subpopulation of infested dogs were evaluated.

**Results:**

Despite the large macro-geographic variation in the dogs screened, there was no consistent difference between East and West Africa in terms of the diversity and numbers of ticks. The highest and lowest numbers of ticks were found in Nigeria and Namibia, respectively. Most often, there was a higher diversity of ticks in rural habitats than in urban habitats, although the highest diversity was observed in an urban Uganda setting. With the exception of Namibia, more fleas were collected in rural areas. We identified tick species (including *Haemaphysalis spinulosa*) as well as zoonotic pathogens (*Coxiella burnetti*, *Trypanosoma* spp.) that are not classically associated with companion animals. *Rhipicephalus sanguineus* was the most abundant tick, with a preference for urban areas. Exophilic ticks, such as *Haemaphysalis* spp., were more often found in rural areas. Several multi-host ticks occurred in urban areas. For *R. sanguineus*, housing conditions and additional pets were relevant factors in terms of infestation, while for a rural tick species (*Haemaphysalis elliptica*), free-roaming dogs were more often infested. Tick occurrence was associated to the use of endoparasiticide, but not to the use of ectoparasiticide. The most prevalent tick-borne pathogen was *Hepatozoon canis* followed by *Ehrlichia canis*. High levels of co-parasitism were observed in all countries and habitats.

**Conclusions:**

As dogs share a common environment with people, they have the potential to extend the network of pathogen transmission to humans. Our study will help epidemiologists to provide recommendations for surveillance and prevention of pathogens in dogs and humans.

**Graphical abstract:**

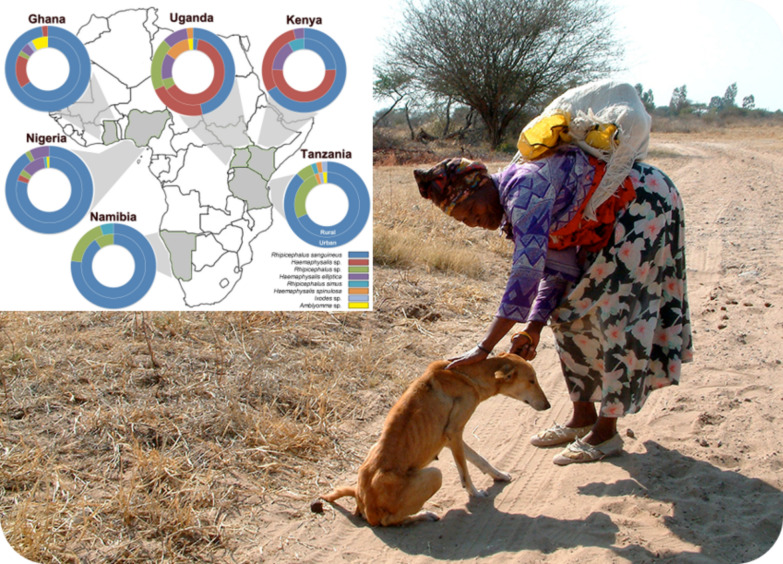

**Supplementary Information:**

The online version contains supplementary material available at 10.1186/s13071-021-05014-8.

## Background

More than half (around) 55% of the world population currently live in urban areas, with estimates for 2050 rising to 68% and close to 90% of this rapid increase taking place in Asia and Africa [1, 2]. In sub-Saharan Africa, expansion of farmland and urbanization—partly due to economic growth—are two of of the most drastic and widespread manifestations of human-driven environmental change. Human-mediated environmental alterations, such as deforestation and establisments of settlements in natural ecosystems, give rise to increased risk of exposure to vector-borne pathogens and new opportunities for novel vector-borne transmission cycles [3]. Green infrastructures are needed for livestock, but they are promoted in urban areas as solutions for a wide range of services, including water management, air quality, recreation services related to biodiversity, among others [4, 5]. However, green (open) spaces near crowded areas are associated with increased human exposure risk to wildlife-associated parasites and pathogens [see 6] and, although reversing exposure to wildlife itself, there is increased exposure through domestic animals [7].

Dogs and cats are implicated in the changing epidemiology of pathogens of public health concern [3, 7]. The role of companion animals in vector-borne diseases in sub-Saharan Africa has not been addressed by the One Health organization in a standardized macro-geographic way. While veterinarians in these countries work hard, their numbers are few, they often work in geographically isolated areas, with limited resources, and they might be hampered by limitations in training. The need has emerged to properly understand urban and rural zoonotic disease risk areas. The socio-economic value of pets has increased in importance in recent decades, especially for sedentary household and farmers. Fleas and ticks are the most common ectoparasites of dogs, and both vector a wide array of vector-borne pathogens that cause diseases such as borreliosis, bartonellosis, ehrlichiosis, rickettsiosis and anaplasmosis. They are both a nuisance and a substantial threat to canines, both directly and indirectly, through the pathogens they transmit. While the former pertains to clinical signs of physical damage, such as wounds and rashes due to bites, the latter often relates to tick-borne diseases in wild and domestic dogs [7–9].

Local tick and flea abundances depend in a multifaceted way on the presence of multiple hosts in a suitable ectoparasite habitat. Implementation of effective measures to control (zoonotic) diseases, such as the establishment of proper treatment strategies and prevention, relies on the elucidation of pathogen and reservoir hosts in a given area [3]. Given the importance of small companion animals, we initiated a multi-country surveillance study on flea and tick communities and their pathogens in sub-Saharan Africa. As part of the African Small Companion Animal Network **(**AFSCAN), which focuses on enhancing companion animal veterinary care across Africa through the creation of a sustainable veterinary network for Africa, we have attempted to identify the vector-borne pathogens and determine and ectoparasite status of dogs in both rural and urban areas in six African AFSCAN countries: Ghana, Kenya, Nigeria, Tanzania, Uganda and Namibia. Based on the collection of biological samples (ectoparasites, blood and serum) we attempted to answer the following questions: (1) To which extent do ectoparasite and their pathogen communities vary macro-geographically? (2) Are parasite communities in urbanized areas similar to those of rural areas? (3) Which additional extrinsic risk factors and host characteristics (age, sex, health status, anti-parasite treatment) are related to ectoparasite infestations and pathogen prevalence (within the group of ectoparasite-infested animals)?

## Methods

### Study design and site

**Fig. 1:**
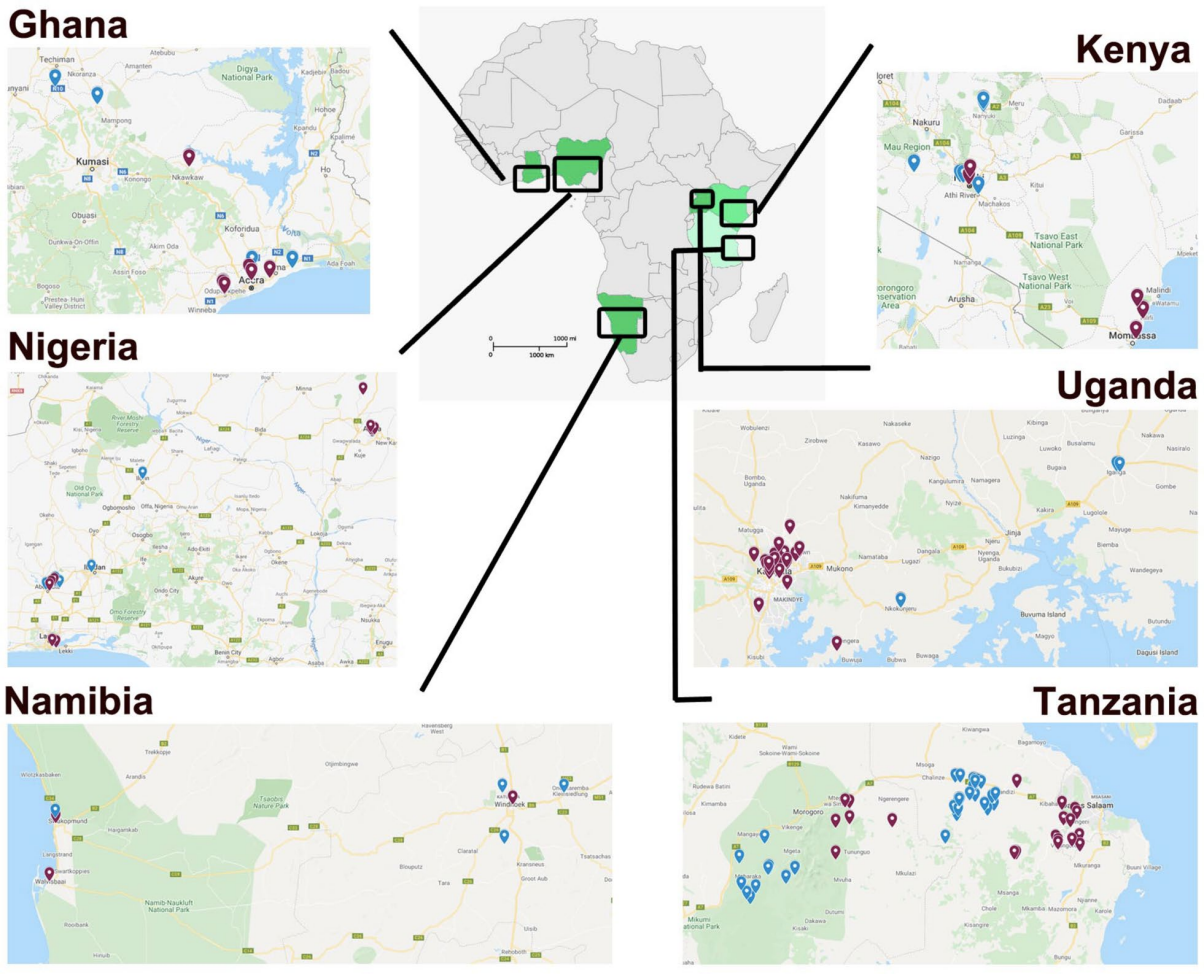
Overview of the sampling locations in the six African countries (Ghana, Kenya, Nigeria, Tanzania, Uganda and Namibia). Locations given in blue and red indicate rural and urban habitats, respectively

This was a multi-site field survey to establish the current community of the most important dog (*Canis familiaris*) ectoparasite species (ticks and fleas) and vector-borne pathogens. Approximately 100 ectoparasite-infested dogs per country (Ghana, Kenya, Nigeria, Tanzania, Uganda and Namibia) were screened and sampled in urban and rural habitats (Fig. 1). In general, urban areas are defined as cities with a large population (> 20,000 inhabitants) and an extensive housing infrastructure (mainly offices, markets), an elaborate network of transportation and access to piped water, modern housing and electricity. Rural areas, on the other hand, are sparsely populated, the infrastructure and housing are poor and in most cases there is no piped water, no electricity and a poor public transportion infrastructure. The main activities in rural areas are associated with agriculture, and dog owners rarely give veterinary care to dogs. All sampling and treating of dogs in the study occurred during the rainy season, except for Namibia (for timing of sampling, see Additional file 1: Fig. S1).

Two regions were sampled in Ghana: the Greater Accra region (GAR) in the south, and Akumadan in the Ashanti region of the country. In GAR, samples were obtained from veterinary clinics in Accra (East Legon, Haatso-Atomic, Dome, Madina) and Tema metropolis. In Akumadan, the settlements from which the pets were brought were Nkwakwaa, Asempanaye, Asuosuo and Afrancho; these are rural and agricultural areas with moist semi-deciduous forest and thick vegetation cover and undergrowth. In Kenya, the urban areas sampled were Nairobi (capital city) and Mombasa (coastal urban town); the dogs sampled here were well kept and had access to housing, good welfare facilities and veterinary care. The rural area (Narok) sampled in Kenya was a pastoral area where dogs accompany livestock to grazing areas and have the potential to interact with wild animals. In Uganda, the two urban areas sampled were located around the capital city of Kampala and Wakiso district, and the two rural areas were located in Mbarara municipality in the western region of Uganda and Iganga municipality in the eastern region. Wakiso district encircles Kampala city, forming peri-urban parts of the capital city. Most of the dogs in Wakiso and Kampala lived in fenced houses for purposes of either companionship or security. In comparison, the rural area of Mbarara district is located in the cattle corridor and dogs there have a wide area of farm land to roam as they herd and guard cattle, with possibility of interacting with wildlife, while the rural area of Iganga is an agricultural base for production of cereals including millet, maize, beans, groundnuts and rice, and dogs guard homes and often follow the owners to the farmed areas and gardens. In Nigeria, one urban and one rural setting was sampled from each of the northcentral and southwest geopolitical division of Nigeria. The rural areas were areas occupied mainly by farmers with no amenities. In Tanzania, the urban areas selected were in the region of Morogoro, and the rural areas were located between Morogoro and Dar es Salaam, the capital. The urban and rural areas selected in Namibia were located between Walvisbaai and Hettiesbaai, in the eastern part of the country bordering the Atlantic Ocean, with the urban sites situated more in the center of the cities whereas the rural areas were more inland.

In order to incentivize owners to provide animals for sampling, sampling was performed in parallel with providing free rabies vaccination and free ectoparasite prevention concomitant therapy. Field assessment screening efforts involved obtaining owner consent and gathering animal details, performing physical examinations, scoring of tick predilection sites and specimen sampling (ectoparasites, blood). Laboratory assessments involved the analysis of samples collected in the field to identify the ectoparasite species collected (ticks, fleas) and the identification of vector-borne parasites. All sampling equipment, data forms, and IDEXX tests (see section Blood collection and processing) were centrally supplied. Molecular analyses (FTA cards [see section Blood collection and processing], identification of vector species and pathogen screening) were performed centrally in the same laboratory (Clinomics, Bloemfontein, South Africa). IDEXX kits were locally interpreted.

### Inclusion criteria

In urban areas, each investigator established a link with a veterinary practice, and privately owned dogs visiting the veterinarian were sample during the visit. In rural areas, where most dogs were likely to be free-roaming and/or community-owned dogs, sampling was performed based around the rabies vaccination/ectoparasite control provision. No sampling was performed at animal shelters. No restriction to breed or age was made. For each registered dog, sex, age, weight, body condition score (5-point-scale: very thin [1], underweight, ideal, overweight, obese [5]), housing (free-roaming, yard, indoor), parasiticide treatment (ectoparasiticide and deworming drugs) and presence of other companion animals were recorded using a standardized data capture form (see Additional file 2: Capture form).

### Ectoparasite burden assessment and collection

Seven different body areas of each dog were systematically screened for ticks, with a burden score assigned individually to each body area : 0 indicating the absence of ticks and 1, 2 and > 3 indicating 1, 2 and > 3 ticks, respectively. For each dog the cumulative number of ticks was determined (the scores of all body areas were summed), and for the statistical analyses, three categories were created: the ‘No’ (cumulative number of ticks: 0), ‘Light infestation’ (1–3), ‘Moderate infestation’ (4–10 ticks) and ‘Severe infestation’ (> 11) categories. For fleas, an estimate for the entire animal was obtained as follows: ‘No’ (0 fleas), ‘Light infestation’ (1–10 fleas), ‘Moderate infestation’ (11–50 fleas) and ‘Severe infestation’ (> 50 fleas). Up to 30 ticks were collected from each animal and placed into a plastic tube with screwcap that contained 70% ethanol. As many fleas as possible were collected from the animal and placed into the same collection jar as the ticks (i.e. one jar per animal).

### Blood collection and processing

Whole blood samples were collected using a syringe and the appropriate needle. Blood was used for the preparation of a Whatman® FTA® card (GE Healthcare, Chicago, IL, USA) on which blood was preserved for DNA analyses. Serum was used for the IDEXX’s 4Dx Plus kits (IDEXX Laboratories, Inc., Westbrook, ME, USA) screening tool for *Ehrlichia canis/ewingii*, *Anaplasma phagocytophilum/platys*, *Borrelia burgdorferi* and *Dirofilaria immitis* following the manufacturer’s manual. Serum was obtained by centrifuging the collected blood after clotting in plain collection tubes; the serum was stored frozen at − 20 °C in plastic screw-cap tubes for future research.

### Vector-borne pathogen identifications

Blood samples were shipped using FTA card technology. The cards were punched (diameter of punches: 3 × 5 mm) and subjected to DNA isolation procedures. Per dog, five ticks (or < 5 if fewer ticks were found) and 5 fleas (or < 5 if fewer fleas were found) were randomly sampled from the jar for molecular identification using multiplex PCR. Ticks and fleas collected from each animal were pooled separately for DNA isolation. These samples were homogenized by bead-bashing before being processing using the MagMAX™ DNA Multi-Sample Ultra Kit (Thermo Fisher Scientific, Waltham, MA, USA) according to the manufacturer’s specifications and eluted with 100 µL elution buffer.

Blood samples and ectoparasites were subsequently screened using PCR techniques for the presence of following tick-borne pathogens: *Babesia rossi*,* Babesia canis*,* E. canis*,* Ehrlichia chaffeensis*,* A. platys*,* A. phagocytophilium*,* Rickettsia conorii*,* Rickettsia africae*,* Coxiella burnetti* and* Hepatozoan canis*. Blood samples were additionally screened for a mosquito-borne (*Dirofilaria immitis*) and a tsetse fly-borne (*Trypanosoma* spp.) pathogen*.*

The isolated DNA (5 µl) served as template in 15-µl hydrolysis probe-based multiplex quantitative PCR (qPCR) assays using the Luna® Universal Probe qPCR Master Mix (New England Biolabs Inc., Ipswich, MA, USA) to detect the target of interest according to the host (canine blood) and sample type (ticks, fleas). A universal thermal cycling program was used for all multiplexes, excluding the *Dipylidium caninum* plex which had an extended elongation time due to the longer expected amplicon size. The targets and their respective DNA templates are shown in Table 1.

**Table 1:** Overview of the targets and their respective DNA templates used in multiplex qPCR assay screenings

Target	Canine blood	Tick	Flea	Limit of detection (copies/PCR)	References
*Babesia rossi*	X	X		5	[24]
*Babesia canis*	X	X		5	[24]
*Ehrlichia canis*	X	X		5	[25]
*Ehrlichia chaffeensis*	X	X		5	[26]
*Anaplasma platys*	X	X		16	[27]
*Anaplasma phagocytophilum*	X	X		9	[28]
*Rickettsia conorii*	X	X		8	[29]
*Rickettsia africae*	X	X		8	[29]
*Coxiella burnetti*	X	X		8	[30]
*Hepatozoon canis*	X	X		5	In-house^b^
*Dirofilaria immitis*	X			8	[31]
*Trypanosoma* spp.^a^	X			5	[32, 33]
*Bartonella henselae*			X	5	[34]
*Mycoplasma haemofelis*			X	16	[35]
*Babesia felis*		X		8	In-house^b^
*Dipylidium caninum*			X	8	[36]

^a^viz*. Trypanosoma vivax, T. congolense, T. evansi* and *T. brucei*

^b^Hydrolysis probe was designed in-house (Clinomics, Bloemfontein, South Africa)

All extracted DNA samples were subjected to a first-round screening using a positive extraction control to assess DNA isolation and an internal amplification control to assess template-derived inhibition of the PCR. In cases where neither the internal amplification control nor any other targets were detected, the samples were diluted 1:1 using 10% Chelex Resin (Bio-Rad laboratories, Hercules, CA, USA). The results were obtained and analyzed using QuantStudio™ Real-Time PCR software (Thermo Fisher Scientific) to determine which samples had detectable levels of target DNA. All qPCR runs included a DNA-negative extraction control, a host-negative control which indicated that the assays did not detect host DNA, a no-template control and a positive control.

Primers and probes (*H. canis*, *D. immitis* and *Babesia felis*) were designed using Geneious (http://www.geneious.com/) and validated in silico using sequence data available on GenBank (Table 2).

**Table 2:** Overview of primers and probes used for the in-house qPCR-screenings of three pathogenic agents

Target	In-house forward primer	In-house reverse primer	In-house hydrolysis probe
*Hepatozoon canis*	GGCAGTGACGGTTAACGGGGG	GCACCAGACTTGCCCTCCAATTG	CCGGAGAGGGAGCCTGAGAAACGG
*Dirofilaria immitis*			CTTTGGAATATGTGTTTTTTTGGAGAGCCCTC
*Babesia felis*	AAGAAGCTCGTAGTTGAATTTCTGCC	GAGAAGCCGAAGCAACACAAATCCAG	TGCGTTTTCCGACTGGCTTGGCA

For all PCRs, the final forward and an reverse primer concentrations were 400 nM. The final probe concentration was 200 nM

### Vector identification

The PCRs were performed using the Q5® Hot Start High-Fidelity 2× Master Mix (New England Biolabs, Inc.) in 10-µl reaction volumes containing 2 µl of DNA isolated from tick/flea using primers 5′-AAAGATGACCAAACTTGATCATTTAGAGG-3 and 5′-TCGATGAAGAACGCAGCCAGCT-3′ at a final concentration of 500 nM each which amplifies the internal transcribed spacer 1 (ITS1) region of the ticks and fleas. Thermal cycling entailed a polymerase activation step at 98 °C for 2 min; then 30 cycles of 98 °C for 10 s, 65 °C for 20 s and 72 °C for 75 s; with a final extension step at 72 °C for 5 min. The PCR products were sequenced and analyzed using the Basic Local Alignment Search Tool (BLAST) for identification.

For ticks which could not be identified using this sequenced region, primers which amplified the 16S ribosomal mitochondrial DNA (mtDNA) of the ticks were used [3]. The reactions were performed using the Platinum™ SuperFi II PCR Master Mix (Thermo Fisher Scientific) in 10-µl reactions containing 2 µl of DNA isolated from ticks with the primers at 500 nM final concentration each. Thermal cycling entailed a polymerase activation step at 98 °C for 2 min; then 30 cycles 98 °C for 10 s, 60 °C for 45 s and 72 °C for 30 s; with a final extension step at 72 °C for 5 min.

### Statistical analysis

For the tick- and flea-infested subpopulations, the proportions of infested dogs per ectoparasite taxon and their infestation intensities were compared between countries (Ghana, Kenya, Nigeria, Tanzania, Uganda and Namibia) and urbanization level (urban vs rural), as well as the proportion of pathogen-infected ectoparasite batches. We emphasize that for each country, urban and rural settings are different (see descriptions above), meaning that a generalized continent-wise comparison ‘urban versus rural’ has little epidemiological relevance. For the flea- and tick-infested dogs (overall number: 584), the abovementioned proportions and intensities were included as response variables in models with the following explanatory variables: the individual’s intrinsic (age, sex, body condition, deworming drugs and ectoparasiticide) and extrinsic risk factors (pet density and housing conditions). For the proportion of pathogen-infected hosts (i.e. based on blood and serum screenings) the pooled sample (i.e. all sampled individuals, irrespective of the type of ectoparasite they were infested with) was considered (overall number: 601), assuming that past infections not necessarily relate to current ectoparasite status. For this purpose, generalized estimation equation models (GEE) were fitted to the data [see 10], taking into account the statistical dependence of observations in the same areas. The residuals for burden categories and pathogen proportions were assumed to follow a binomial distribution (logit-link, in ordinal and logistic regression, respectively). Because of the limited amount of independent data as well as the high number of tests on the same set of plots, the following model restrictions were imposed on models that included extrinsic and intrinsic risk factors: (i) no interaction terms among the main explanatory variables were fitted as adding these to the model would lead to (almost) saturated models and reductions in statistical power for each of the tests; and (ii) only those variables that were highly significant (*P* < 0.01) were considered to be the main result in the Discussion section and Abstract. A variable that explained part of the variation, though in a less significant way (*P* < 0.05), was left in the models to correct for its confounding effect and to provide further inspiration for future studies. For all analyses, a stepwise backward selection procedure was used to select the best model. At each step we excluded the fixed factor with the highest non-significant *P*-value (*P* > 0.05), re-ran the model and examined the *P*-values of the fixed factors in the reduced model. Model reduction continued until only significant factors (*P* < 0.05) and their lower order interaction terms were left [11]. For the statistical comparison of the parasite community, Fisher’s exact tests were executed whereby the species distribution (in the population of parasitized individuals) was compared between habitat types (urban vs rural) and countries. In addition, the Shannon diversity index was computed [12]. All prevalence estimates are reported as the mean ± standard error (SE). All data management and statistical analyses were performed in SAS v 9.3 (SAS Institute, Cary, NC, USA).

## Results

### Ectoparasites

Of all infested dogs examined (*N* = 584), 95.4% had ticks and 51.9% fleas (47.3% were co-infested with both ticks and fleas). In total, 13 tick and three flea taxa were identified based on the ectoparasite’s DNA. Higher ectoparasite diversity was found in rural areas compared to urban areas (see Shannon index, Table 3), with the exception of Uganda and Namibia. The highest and lowest diversity of ectoparasites was found in rural Ghana (Shannon index:  1.60; 10 different taxa identified in 42 infested individuals) and urban Nigeria (Shannon index: 0.44; 4 taxa in 51 individuals), respectively. Ectoparasite communities (fleas and tick species; Additional file 1: Table S1) significantly differed among each country (Fisher’s exact tests; for all pair-wise comparisons among countries *P* < 0.001). In the following sections we report on the dogs’ geographic occurrence and extrinsic and intrinsic exposure risk factors, all of which are related to ectoparasite prevalence and infestation intensity.

**Table 3:** Tick and flea prevalence and intensity in infested dogs of six African countries

Ticks and Fleas	Overall prevalence (%)	Tanzania (%)			Kenya (%)			Uganda (%)			Nigeria (%)			Ghana (%)			Namibia (%)		
		Rural	Urban	*P*	Rural	Urban	*P*	Rural	Urban	*P*	Rural	Urban	*P*	Rural	Urban	*P*	Rural	Urban	*P*
**Ticks**																			
* Rhipicephalus sanguineus*	67.5	71.1	91.5	ns	27.6	61.5	**	2.3	47.5	**	94.7	96.1	ns	78.4	85.7	*	90.3	83.3	ns
* R. appendiculatus*	0.4	0.0	0.0		0.0	0.0		2.3	2.5		0.0	0.0		0.0	0.0		0.0	0.0	
* R. simus*	2.0	0.0	2.1		9.2	0.0		0.0	0.0		1.8	0.0		0.0	0.0		0.0	5.6	
* R. microplus*	0.2	2.2	0.0		0.0	0.0		0.0	0.0		0.0	0.0		0.0	0.0		0.0	0.0	
* R. senegalensis*	0.2	0.0	0.0		0.0	0.0		0.0	0.0		0.0	2.0		0.0	0.0		0.0	0.0	
* Rhipicephalus* spp.	6.7	26.7	6.4	**	0.0	0.0		2.3	17.5	*	3.5	0.0		2.7	0.0		9.7	19.4	ns
* Haemaphysalis elliptica*	6.5	0.0	0.0		18.4	3.9	*	11.4	10.0	ns	8.8	7.8		5.4	0.0		0.0	0.0	
* H. leachi*	0.6	0.0	0.0		0.0	0.0		0.0	0.0		5.3	0.0		0.0	0.0		0.0	0.0	
* H. spinulosa*	1.5	2.2	2.1		0.0	0.0		11.4	2.5	ns	0.0	0.0		0.0	0.0		0.0	0.0	
* Haemaphysalis* spp.	17.3	0.0	0.0		56.6	26.9	*	54.6	22.5	**	3.5	0.0		18.9	2.0	*	0.0	0.0	
* Amblyomma variegatum*	0.4	0.0	0.0		0.0	0.0		0.0	0.0		1.8	0.0		2.7	0.0		0.0	0.0	
* Amblyomma* spp.	0.9	2.2	0.0		0.0	0.0		2.3	0.0		0.0	0.0		8.1	0.0		0.0	0.0	
* Ixodes* sp.	0.6	2.2	2.1		0.0	0.0		0.0	0.0		0.0	0.0		2.7	0.0		0.0	0.0	
Tick	95.5	100.0	100.0	ns	94.7	88.5	ns	81.8	95.0	ns	100.0	100.0	ns	97.3	87.8	ns	100.0	100.0	ns
Intensity		6.8	21.2	ns	22.5	13.0	ns	12.2	19.2	ns	28.1	49.0	*	8.1	46.3	*	19.4	4.7	ns
Shannon index		0.9	0.5	*	1.2	0.8	***	1.2	1.4	***	0.8	0.4	ns	1.2	0.1	**	0.3	0.7	ns
**Fleas**																			
* Ctenocephalides felis*	53.7	71.1	51.0	ns	75.4	48.2	ns	85.1	41.7	***	52.5	5.9	***	53.3	45.8	ns	12.5	28.6	ns
* Echidnophaga gallinacea*	0.2	0.0	0.0		0.0	0.0		0.0	0.0		0.0	0.0		3.3	0.0		0.0	0.0	
* Echidnophaga* sp.	3.7	0.0	2.0		8.7	0.0		0.0	0.0		0.0	0.0		6.7	2.1		0.0	20.0	***
Flea	55.6	71.1	51.0	*	76.8	48.2	**	85.1	41.7	***	52.5	5.9	***	56.7	47.9	*	12.5	45.7	**
Intensity		6.45	31.8	*	13.6	40.0	*	2.3	5.0		0.0	0.0		9.5	14.3	ns	0.0	0.0	
Shannon index		0.00	0.2	ns	0.3	0.0	ns	0.0	0.0		0.0	0.0		0.5	0.2	ns	0.0	0.7	ns
Co-infestation	47.3	71.7	50.9	**	71.1	37.0	**	68.0	36.0	**	52.6	5.9	*	54.8	35.7	**	12.5	45.5	***
Shannon index^a^		1.25	1.0	ns	1.5	1.1	**	1.3	1.6	***	1.2	0.4	***	1.6	0.8	**	0.5	1.3	*
Investigated dogs (*N*)	584	46	53		76	27		50	50		57	51		42	56		32	44	

**Fig. 2:**
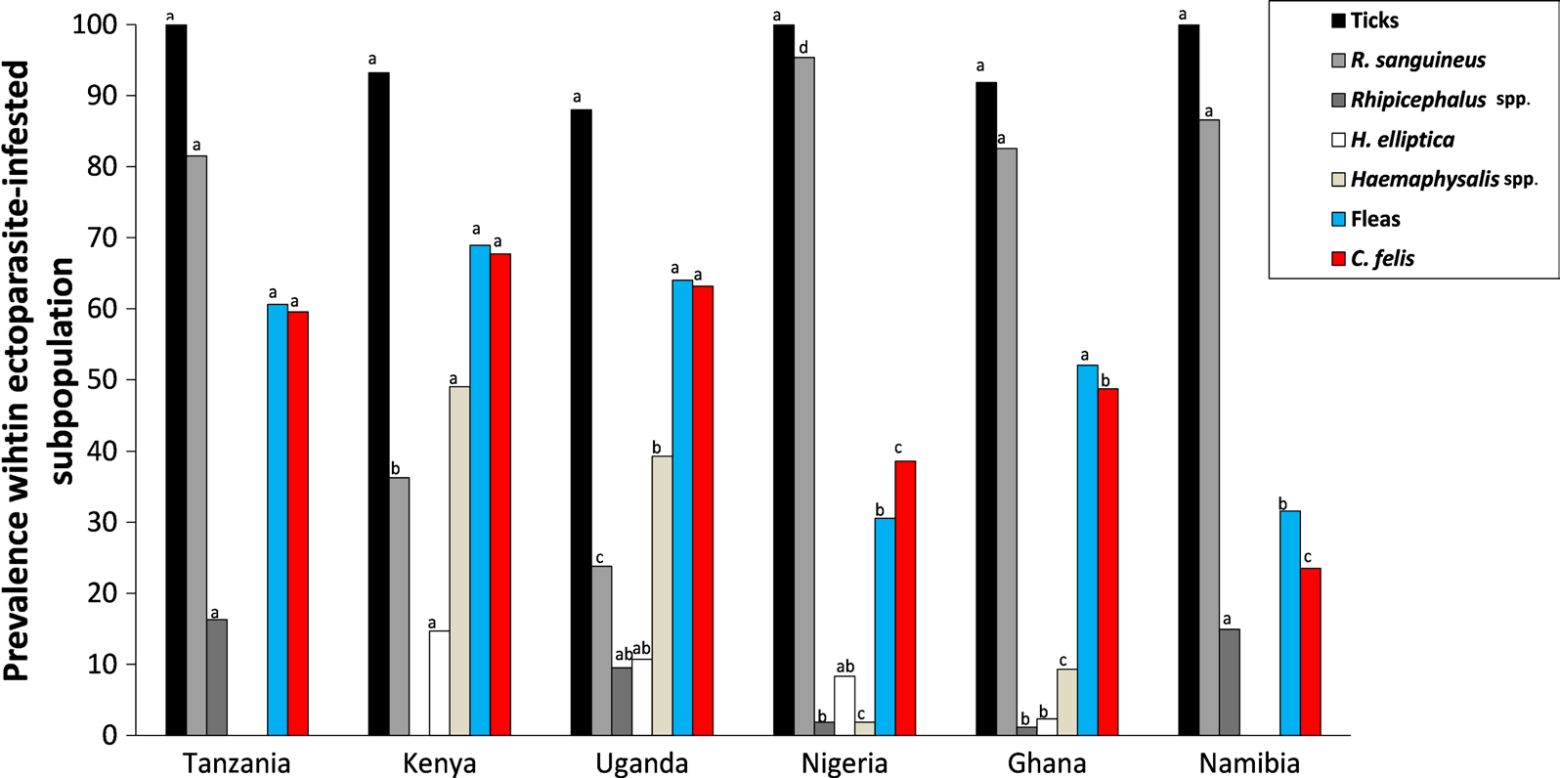
Macro-geographic variation in ectoparasite prevalence. Percentages within the population of infested dogs parasitized with the most common tick (black and gray shading) and flea (red and blue shading) taxa (overall prevalence per taxon > 5%; see Table 3). For each taxon, the same letters above columns indicate that the the contrast between countries is not statistically different from zero

For each dog, a single extraction was made of a pooled set of ticks and/or fleas that was subsequently screened for the presence of DNA belonging to a particular tick and flea species. Next, the percentage of extracts (i.e. dogs) containing DNA of a specific taxon was derived, within the population of infested dogs. For statistical outcomes on pairwise macrogeographic differences, see Fig. 2

***P < 0.001, **P < 0.01, *P < 0.05, ns (not-significant) *P* > 0.05

Habitat differences (rural vs urban) are investigated for countries with a presence of at least 10% in one of its habitats

^a^As a measure of species diversity, a Shannon diversity index and accompanying significance level of Fisher’s exact test are provided

### Tick infestation

#### Prevalence

Within the subpopulation of ectoparasite-infested dogs, ticks were more often found than fleas (Table 3). Consequently, the among-country variation in tick prevalence was low and contrasts between urban and rural areas were small (all *P*-values > 0.05). Literally all examined dogs of Tanzania, Nigeria and Namibia were infested with at least one tick (prevalence 100%), which differed from other locations that showed a higher proportion of flea-infested-only dogs. Prevalences of co-infested individuals (fleas and ticks) significantly varied among locations (range: 5.9–71.7%; *χ*^2^ = 33.85; *df* = 5; *P* < 0.001; Table 3), with the number of co-infested animals overall generally being higher in rural areas (Logit_rural-urban_ = 0.97 ± 0.18; *Z* = 29.70; *P* < 0.001), except in Ghana (no difference; *P* = 0.059) and Namibia where the rural prevalence was lower (Rural < Urban: Logit_rural-urban_ = − 1.76 ± 0.61; *Z* = 8.24; *P* < 0.004). We refer to Fig. 2 for pairwise-comparisons between countries.

#### Community

**Fig. 3:**
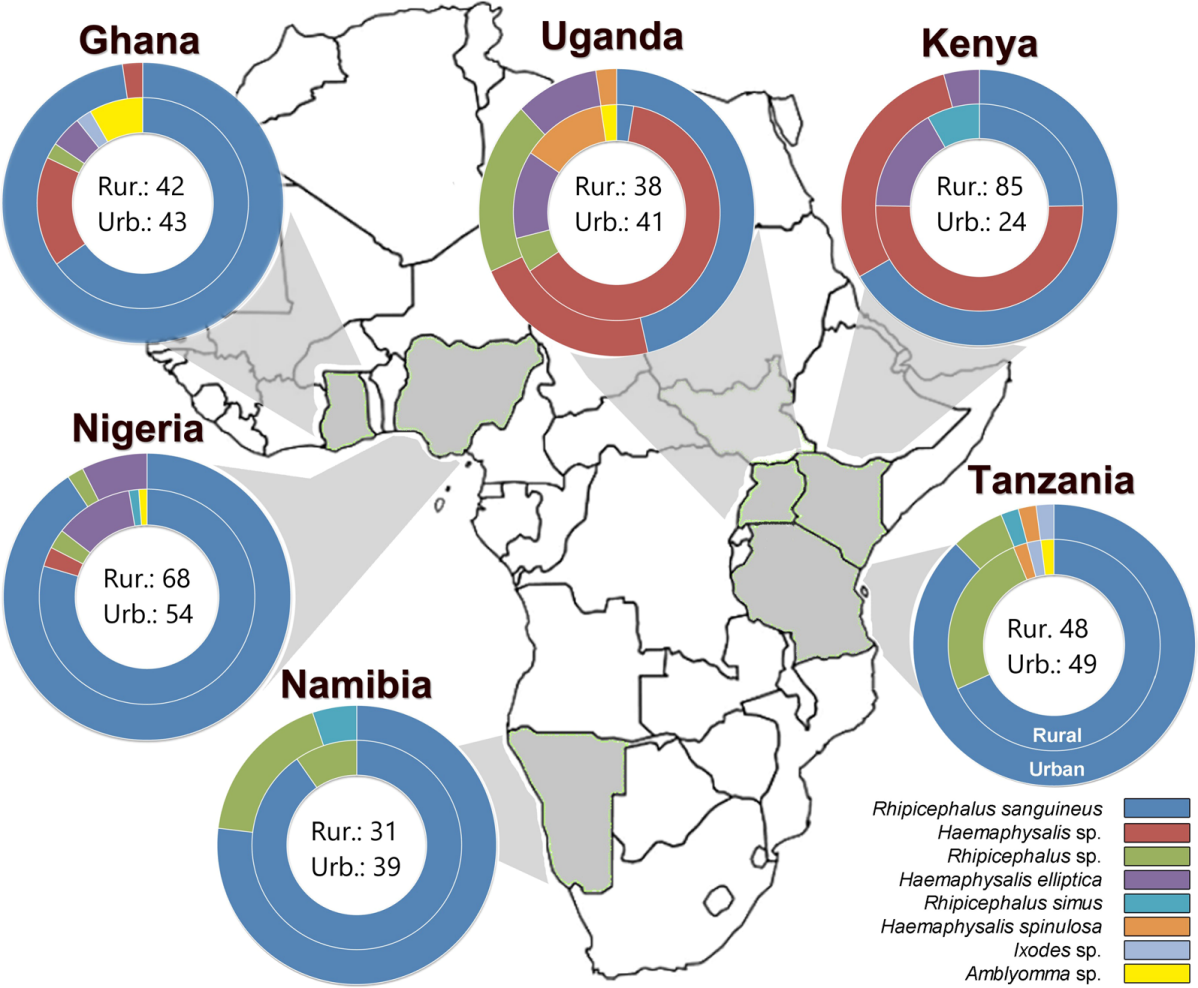
Graphical overview of the tick communities found in urban and rural areas of the six African countries participating in the AFSCAN project (see Additional file 1: Table S1 for raw data). Numbers represent the PCR signals allocated to a tick taxon in the infested dogs. Per dog, DNA was extracted from a pooled set of ticks prior to carrying out the PCR analysis.

With respect to the tick’s community (based on all DNA identifications obtained from the pool of ticks of each dog; see Fig. 3 and Additional file 1: Table S1), the Shannon diversity index was higher in rural areas, with the exception of Namibia (rural: 0.32; urban: 0.66) and Uganda (rural: 1.21; urban: 1.40). Differences were found to be statistically significant in all countries (Table 3) but Namibia and Nigeria (Fisher exact test, *P* = 0.05). Also, the between-country variation in tick communities was high, as all countries significantly differed from each other (Fisher exact test pair-wise country comparisons, *P*-values < 0.001).

The most prevalent genus in almost all areas was *Rhipicephalus* (73.5% of all identifications; Fig. 3), but *Haemaphysalis* was the genus most often found in rural Kenya (67.1%) and Uganda (76.3%). Nine different tick taxa were successfully identified at the species level. *Rhipicephalus sanguineus* was by far the most dominant in the pool of identified ticks (64.5%). Significant proportions of *Haemaphysalis elliptica* within the tick community were found in Kenya, Uganda, Nigeria and rural Ghana. In Uganda, nine *Haemaphysalis spinulosa*-infested dogs were sampled (rural: 13.2%; urban: 9.8% of identifications). A large proportion of ticks could only be identified at genus level, which was especially the case for *Haemaphysalis* spp*.* collected in rural areas. Less than 1% of the tick-infested dogs sampled contained DNA that belonged to the genera *Amblyomma* and *Ixodes*. Co-infestations, i.e. more than one species feeding on the same host individual, were observed in 8.9% of tick-infested dogs, and was dominated by the co-occurrences with *R. sanguineus* (Additional file 1: Table S2).

#### Infestation intensity

The following insights were obtained regarding tick intensity (the ectoparasite loads in tick-infested individuals). First, the among-country variation was high (*χ*^2^ = 82.18; *df* = 5; *P* < 0.001), with the proportion of the subpopulation having intermediate to high loads ranging from 4.7 (Namibia) to 49.0% (Nigeria). In Nigeria, the cumulative Logit_rural-urban_ = 0.77 ± 0.38 (*Z* = 3.97; *P* = 0.046) and in Ghana the cumulative Logit_rural-urban_ = 1.01 ± 0.45 (*Z* = 5.11; *P* = 0.02). Contrasts in infestation intensities (rural vs urban) for all other countries did not significantly differ from zero.

#### Ecological correlations

Analyses were carried out for those country–taxa combinations for which at minimum two countries had a 10% prevalence in a given habitat type (see Table 3). As each taxon contains a different set of countries, comparisons of outcomes between taxa should not be generalized. As mentioned in the Methods section, due to the many tests performed on the same datasets, only factors that significantly explain the variation at* α*-level = 0.01 are discussed in the following sections; however, covariates with *P* < 0.05 remained in the model for their potential confounding effects, as well as a source of inspiration for future research.

In addition to the previously mentioned differences between countries and level of urbanization, the following covariates explained part of the variation in infestations. For *R. sanguineus* loads and prevalence, housing conditions mattered (*χ*^2^ ≥ 48.00; *P* < 0.0001) in that the highest loads were observed in yard dogs. Yard dogs were more often infested (Logit_free-yard_ = − 0.71 ± 0.36; *Z* = 9.91; *P* = 0.048) than free-roaming ones, and carried significantly higher loads (Logit_free-yard_ = − 0.86 ± 0.27; *Z* = 9.93; *P* = 0.0016). The lowest *R. sanguineus* prevalence and load were observed in dogs that were kept indoors. The housing conditions did not show any associations in the other tick taxa, except for *H. elliptica*, with the free-roaming dogs being far more often and heavily infested than the dogs kept indoors (Logit_indoor-free_ = − 1.91 ± 0.69; *Z* = 7.63; *P* = 0.0058). The influence of intrinsic characteristics (age, body condition and sex) varied among tick taxa, but were found to be less important than the effect of deworming (see below). In *Rhipicephalus* spp*.* (i.e. in the cluster of unidentified ticks belonging to the genus *Rhipicephalus*), female dogs were more often infested (Logit_female-male_ = 0.76 ± 0.27; *Z* = 7.81; *P* = 0.005) and body condition showed a positive association with tick prevalence and loads (Logit = 0.60 ± 0.21; *Z* = 8.29; *P* = 0.004). In contrast, tick loads in *R. sanguineus* were negatively correlated with body condition (Logit = − 0.40 ± 0.14; *Z* = 8.27; *P* = 0.004). With regard to deworming drugs, for *Rhipicephalus* sp. and *H. elliptica*, dogs that never received deworming drugs were less likely to be infested (see Table 4 for contrasts). In contrast, for *Haemophysalis* spp. (i.e. in the cluster of unidentified ticks belonging to the genus *Haemophysalis*), the group that received a drug > 6 months previously had significantly more ticks than the recently treated ones (within 6 months), but also more than the untreated dogs. For *R. sanguineus*, no association with deworming drugs were found. With regard to other extrinsic exposure risk factors, in addition to habitat and macro-geographic sources of variation, in environments with more dogs around we found slightly fewer *H. elliptica*-infested dogs (Logit = − 0.21 ± 0.08; *Z* = 6.95; *P* = 0.008), but more *Haemophysalis* sp.-infested ones (Logit = 0.16 ± 0.05; *Z* = 10.51; *P* = 0.0012).

**Table 4:** Ecological models for ectoparasite prevalence and loads in infested dogs

Covariate	*R. sanguineus *: all countries		*Rhipicephalus* sp.: Ta., Ug., Na.		*H. elliptica*: Ke., Ug.		*Haemaphysalis.* sp.: Ke., Ug., Gh.		*C. felis*: all countries	
	Prevalence^a^	Load^b^	Prevalence	Load	Prevalence	Load	Prevalence	Load	Prevalence	Load
Housing conditions^c^	F/Y: − 0.71 ± 0.36	− 0.86 ± 0.27**			F/Y: 0.43 ± 0.29	0.13 ± 0.35				
	I/Y: − 0.96 ± 0.41*	− 1.80 ± 0.30**			I/F:-1.89 ± 0.65**	− 1.91 ± 0.69**				
Age (months)										
Sex										
Female vs male			0.72 ± 0.27**	0.76 ± 0.27**						
Body condition	− 0.37 ± 0.18*	− 0.40 ± 0.14**	0.57 ± 0.21**	0.60 ± 0.21**		0.35 ± 0.17*				
Deworming^d^										
< 1 month			2.08 ± 0.42***	2.15 ± 0.44**	0.56 ± 0.52	1.95 ± 0.66**	− 0.29 ± 0.33	− 0.30 ± 0.33	− 0.87 ± 0.35*	− 1.24 ± 0.39**
1–6 months			2.11 ± 0.45***	2.17 ± 0.42**	3.57 ± 0.84***	4.27 ± 0.76**	− 0.62 ± 0.33	− 0.62 ± 0.32	− 1.19 ± 0.39**	− 1.28 ± 0.42**
> 6 months			1.46 ± 0.53**	1.42 ± 0.53**	− 0.85 ± 0.77	− 0.18 ± 0.83	1.03 ± 0.31**	1.22 ± 0.28**	− 0.48 ± 0.40	− 0.01 ± 0.39
Number of dogs around			− 0.13 ± 0.05*	− 0.13 ± 0.05*	− 0.21 ± 0.08**	− 0.42 ± 0.12*	0.16 ± 0.05**	0.14 ± 0.04*		

Parameter estimates (± empirical standard error) from eneralized estimation equations (GEEs) that model the tick and flea species’ prevalence (levels: 0, 1) and infestation loads (levels: absent, low, intermediate, high) for the population of ectoparasite-infested dogs. For a given taxon, only multiple countries with a prevalence of at least 10% for at least one of the habitat types were included (see Table 3). Country and habitat (rural vs urban) contrasts have been omitted from the table, but were included in all analyses. In none of the analyses did ‘ectoparasiticide treatment’ significantly explain tick variation (*P* > 0.05)

Ta(nzania), Ug(anda), Na(mibia), Ke(nya), Ni(geria), Gh(ana)

***P < 0.001, **P < 0.01, *P < 0.05, ' ' *P* > 0.05

^a^Prevalence indicates: model estimates reflect the probability that ectoparasite has level ‘1’; ^b^Load indicates model estimates that reflect the probabilities of tick load levels having higher ordered values, i.e. positive signs indicate higher loads with continuous explanatory variables, or higher than the reference category (when contrast is tested among the levels of categorical explanatory variables)

^c^Housing conditions contrasts include: F/Y (Free-roaming vs Yard); I/Y (Indoor vs Yard); I/F (Indoor vs Free-roaming); ^d^Contrast with group of dogs that have never been treated with a deworming drug

### Fleas

#### Prevalence

Substantial variation was observed for fleas among countries (*χ*^2^ > 56.12; *df* = 5; *P* < 0.003), but it should be emphasized that—due to the dominance of ticks—flea prevalence outcomes are to be interpreted with care and only for the subpopulation of ectoparasite-infested dogs. In particular, dogs from rural areas had significantly more fleas than dogs from urban areas (*χ*^2^ = 29.32; *df* = 1; *P* < 0.001), and this pattern was consistent across almost all countries (range Logit_rural-urban_ = 1.01 [Tanzania], 2.13 [Uganda]; *Z* = 6.09–18.42; *P* = 0.013 to < 0.0001). Namibia was an exception to the rule, with more fleas observed in urban dogs (Logit_rural-urban_ = − 2.05 ± 0.68; *Z* = 9.13; *P* = 0.0025). Further macro-geographical contrasts at the taxon level are shown in Table 3 and Fig. 2. By far, *Ctenocephalides felis* was the most prevalent flea species (overall prevalence: 53.7%). *Echidnophaga gallinacea*-infested individuals were only found in Ghana (3.3%). Unidentified *Echidnophaga* spp*.* were collected from a large proportion of the dogs (20%) tested in urban Namibia. In those dogs with flea co-infestations (3.5% of infested dogs), *C. felis × Echidnophaga* sp. was the most common combination observed (3.1% overall), with the highest occurrence in the rural Kenya setting (9.4%) (Additional file 1: Table S3).

#### Infestation intensity

The among-country variation was high (*χ*^2^ = 23.31; *df* = 5; *P* < 0.001), with the proportion of dogs with intermediate to high loads ranging from 0% (Nigeria and Namibia) to 40.0% (urban Kenya). In Kenya, intensities were significantly higher in urbanized areas (cumulative Logit_rural-urban_ = 1.72 ± 0.71; *Z* = 6.00; *P* = 0.014).

#### Ecological correlations

None of the intrinsic risk factors were associated with *C. felis* flea loads. Dogs that were never treated with deworming drugs tended to have more fleas than dogs that were treated more recently (< 6 months; see Table 4 for contrasts). No additional effects of extrinsic risk factors were found.

### Vector-borne pathogens

DNA of pathogenic agents was detected in the collected blood samples (7 pathogen taxa), ticks (9 taxa) and fleas (3 taxa); serum antibodies against four pathogen genera were also detected. For all biological samples, countries strongly differed in terms of pathogen distributions and prevalence. In contrast to the findings for ectoparasites, habitat differences in the vector-borne pathogens were less consistent and obvious.

#### Host blood

**Fig. 4:**
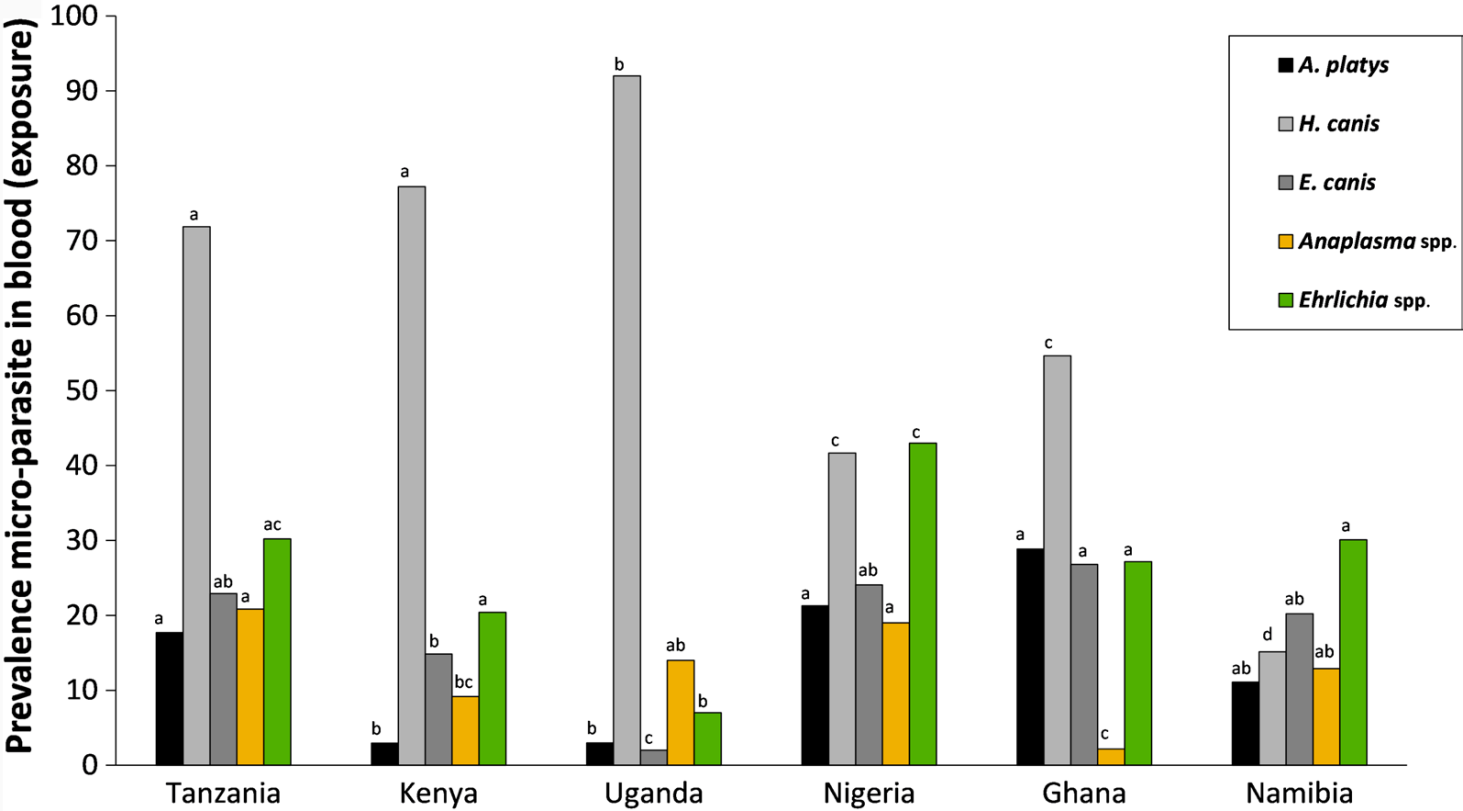
Macro-geographic variation in pathogen (sero-) prevalence in the blood samples collected from dog. Percentages of dogs infected with vector-borne pathogens based on DNA screening (gray shading), and seroprevalence against two taxa (yellow and green shading). For each pathogen, the same letters above columns indicate that the the contrast between countries is not statistically different from zero

*Hepatozoon canis* was the most prevalent pathogen found in the blood samples of 601 dogs (overall 58.6%), but its prevalence varied greatly among country–habitat combinations (range: 8.82–98%; *χ*^2^ = 159.55; *df* = 5; *P* < 0.001). This pathogen was found to be consistently more prevalent in rural areas than in urban areas (*χ*^2^ = 33.83; *df* = 1; *P* < 0.001). This was also the case for *Ehrlichia canis* and *A. platys* in Nigeria. Figure 4 and Table 5 provide an overview of prevalence data and contrasts between countries and/or habitats, respectively.

**Table 5:** Pathogen prevalence in the blood of dogs from six African countries

Pathogen prevalence	Overall	Tanzania (%)			Kenya (%)			Uganda (%)			Nigeria (%)			Ghana (%)			Namibia (%)		
		Rural	Urban	*P*	Rural	Urban	*P*	Rural	Urban	*P*	Rural	Urban	*P*	Rural	Urban	*P*	Rural	Urban	*P*
**Pathogens in blood**																			
* B. canis*	0.0	0.0	0.0		0.0	0.0		0.0	0.0		0.0	0.0		0.0	0.0		0.0	0.0	
* B. rossi*	3.8	0.0	1.9		12.0	3.9		8.0	6.0		3.5	3.9		0.0	1.8		0.0	0.0	
* E. chaffeensis*	0.0	0.0	0.0		0.0	0.0		0.0	0.0		0.0	0.0		0.0	0.0		0.0	0.0	
* A. platys*	14.1	13.6	21.2	ns	4.0	0.0		0.0	6.0		33.3	7.8	**	27.5	29.8	ns	19.3	7.4	ns
* A. phagocytophilum*	0.0	0.0	0.0		0.0	0.0		0.0	0.0		0.0	0.0		0.0	0.0		0.0	0.0	
* R. conorii*	0.0	0.0	0.0		0.0	0.0		0.0	0.0		0.0	0.0		0.0	0.0		0.0	0.0	
* R. africae*	0.0	0.0	0.0		0.0	0.0		0.0	0.0		0.0	0.0		0.0	0.0		0.0	0.0	
* C. burnetti*	0.5	0.0	0.0		2.7	0.0		0.0	0.0		1.8	0.0		0.0	0.0		0.0	0.0	
* H. canis*	58.6	77.3	67.3	ns	85.3	53.9	**	98.0	86.0	ns	56.1	25.5	**	67.5	45.6	*	29.0	8.8	*
* E. canis*	18.5	27.3	19.2	ns	13.3	19.2	ns	0.0	4.0		31.6	15.7	*	25.0	28.1	ns	22.5	19.1	ns
* D. immitis*^a^	2.8	4.6	5.8		1.3	0.0		0.0	0.0		0.0	0.0		7.5	14.0	ns	0.0	0.0	
* Trypanosoma* spp.^a^	0.2	0.0	0.0		0.0	0.0		0.0	0.0		1.8	0.0		0.0	0.0		0.0	0.0	
Individuals (N)	601	44	52		75	26		50	50		57	51		40	57		31	68	
Shannon index		1.0	1.1	ns	1.0	0.8	ns	0.3	0.6	ns	1.3	1.2	**	1.2	1.4	ns	1.1	1.0	ns
**Sero-positive**																			
* Anaplasma* spp.	13.1	20.5	21.2	ns	9.7	7.7		4.0	24.0	**	4.0	20.5	***	0.0	3.9		23.3	7.9	**
* Borrelia* spp.	0.2	0.0	0.0		1.4	0.0		0.0	0.0		0.0	0.0		0.0	0.0		0.0	0.0	
* Ehrlichia* spp.	26.3	31.8	28.9	ns	22.2	15.4	ns	10.0	4.0	ns	54.0	32.0	*	35.0	21.2	ns	40.0	25.4	ns
Heartworm^b^	0.3	0.0	0.0		0.0	3.9		0.0	0.0		0.0	0.0		0.0	1.9		0.0	0.0	
Individuals (*N*)	579	44	52		72	26		50	50		50	50		40	52		30	63	

DNA-based (qPCR) pathogen prevalence in the blood of dogs from urban and rural areas of 6 African countries. Prevalence of 2 additional pathogens—not transmitted by fleas or ticks (*D. immitis* and *Trypanosoma* spp.)—are reported as well. In addition, sero-prevalence of four pathogen genera are given, for which the IDEXX test was used. For statistical outcomes on pairwise macro-geographic differences, see Fig. 4. As a measure of species diversity, a Shannon index has been provided

***P < 0.001, **P < 0.01, *P < 0.05, ns (not-significant) *P* > 0.05

^a^Non-tick-or flea-borne pathogens

^b^Positive in IDEXX test, but all negative in qPCR’s

No further analyses were performed on pathogens that were not detected (*B. canis*,* E. chaffeensis*,* A. phagocytophilum*,* R. conorii*) or occurred in very low numbers (*B. rossi* [3.8%], *C. burnetti* [0.5%], *D. immitis* [2.8%], *Trypanosoma* spp. [0.2%]). Of all dogs that were infected with at least one pathogen (*N* = 434), 30.9% were co-infected with at least two pathogenic agents, with *H. canis × E. canis* (10.1%; Additional file 1: Table S4) being the most prevalent combination. Especially in Ghana and rural Nigeria, co-infections were commonly seen (> 40%). Several individuals (5.1%) had three to four pathogens in their blood. In terms of pathogen community distributions, most country communities greatly differed from each other (Fisher exact tests, all *P*-values < 0.014), with the exception of the pairwise comparisons Namibia versus Nigeria (*P* = 0.21) and Ghana versus Tanzania (*P* = 0.093). Surprisingly, within each country no significant habitat differences (urban vs rural) in pathogen distribution were found (all *P*-values > 0.05).

Seroconversion was detected most often in response to *Anaplasma* spp. (13.1%) and *Ehrlichia* spp. (26.3%). For most of the six countries studied, seroprevalences were higher than their respective pathogen genera traced in the host blood (Fig. 4; Table 5).

#### Ticks

Also in ticks (537 pools screened), *H. canis* (60.5%) was the most prevalent pathogen. The absence of *C. burnetti* in the blood contrasts with the results from analysis of the tick pools (15.1%). The prevalence of *A. platys* (10.6%) was similar to that in the blood, while *E. canis* (overall 5.4%) was less often found. In addition to the highly significant variation among countries (*χ*^2^ > 25.03; *df* = 5; all *P*-values < 0.001), in Nigeria, three pathogens were significantly more prevalent in rural habitats than in urban habitats (Table 6).

**Table 6:** Pathogen prevalence in flea and tick pools collected from infested dogs

Tick and flea pools	Overall	Tanzania (%)			Kenya (%)			Uganda (%)			Nigeria (%)			Ghana (%)			Namibia (%)		
		Rural	Urban	*P*	Rural	Urban	*P*	Rural	Urban	*P*	Rural	Urban	*P*	Rural	Urban	*P*	Rural	Urban	*P*
**Ticks**																			
* B. canis*	0.0	0.0	0.0		0.0	0.0		0.0	0.0		0.0	0.0		0.0	0.0		0.0	0.0	
* B. rossi*	2.4	0.0	0.0		6.9	0.0		0.0	6.5		1.8	3.9		5.1	0.0		0.0	0.0	
* B. felis*	0.6	4.4	0.0		0.0	0.0		0.0	0.0		1.8	0.0		0.0	0.0		0.0	0.0	
* E. chaffeensis*	0.0	0.0	0.0		0.0	0.0		0.0	0.0		0.0	0.0		0.0	0.0		0.0	0.0	
* A. platys*	10.6	20.0	26.0	ns	0.0	0.0		0.0	6.5		29.8	3.9	**	15.4	13.3	ns	0.0	2.6	
* A. phagocytophilum*	0.0	0.0	0.0		0.0	0.0		0.0	0.0		0.0	0.0		0.0	0.0		0.0	0.0	
* R. conorii*	3.7	2.2	0.0		18.1	0.0	**	5.3	6.5		0.0	0.0		0.0	0.0		0.0	2.6	
* R. africae*	2.1	4.4	0.0		1.4	0.0		7.9	0.0		1.8	0.0		7.7	2.2		0.0	0.0	
* C. burnetti*	15.1	11.1	2.0	ns	63.9	0.0	***	15.8	10.9	ns	29.8	2.0	**	0.0	0.0		0.0	0.0	
* H. canis*	60.5	75.6	76.0	ns	80.6	62.5	ns	50.0	41.3	ns	91.2	58.8	***	56.4	46.7	ns	29.0	20.5	ns
* D. immitis*	0.2	0.0	0.0		0.0	0.0		0.0	0.0		0.0	0.0		0.0	2.2		0.0	0.0	
* E. canis*	5.4	2.2	8.0		2.8	0.0		0.0	0.0		12.3	13.7	ns	10.3	6.7	ns	3.2	0.0	
Tick pools (N)	537	45	50		72	24		38	46		57	51		39	45		31	39	
Shannon index		1.2	0.9	ns	1.2	0.0	**	1.0	1.3	ns	1.3	0.9	**	1.2	1.0	ns	0.3	0.6	ns
**Fleas**																			
* B. henselae*	0.8	0.0	0.0		0.0	0.0		0.0	0.0		0.0	0.0		0.0	8.3		0.0	0.0	
* M. haemofelis*	14.2	3.7	16.0	ns	22.6	30.8	ns	12.5	10.0	ns	13.6	0.0		5.9	12.5	ns	0.0	11.8	
* D. caninum*	11.9	3.7	4.0		3.8	0.0		27.5	25.0	ns	31.8	0.0		5.9	4.2		0.0	11.8	
Flea pools (N)	261	27	25		53	13		40	20		22	1		17	24		2	17	
Shannon index		0.7	0.5	ns	0.4	0.0	ns	0.6	0.6	ns	0.6	0.0		0.7	1.0	ns	0.0	0.7	

**Fig. 5:**
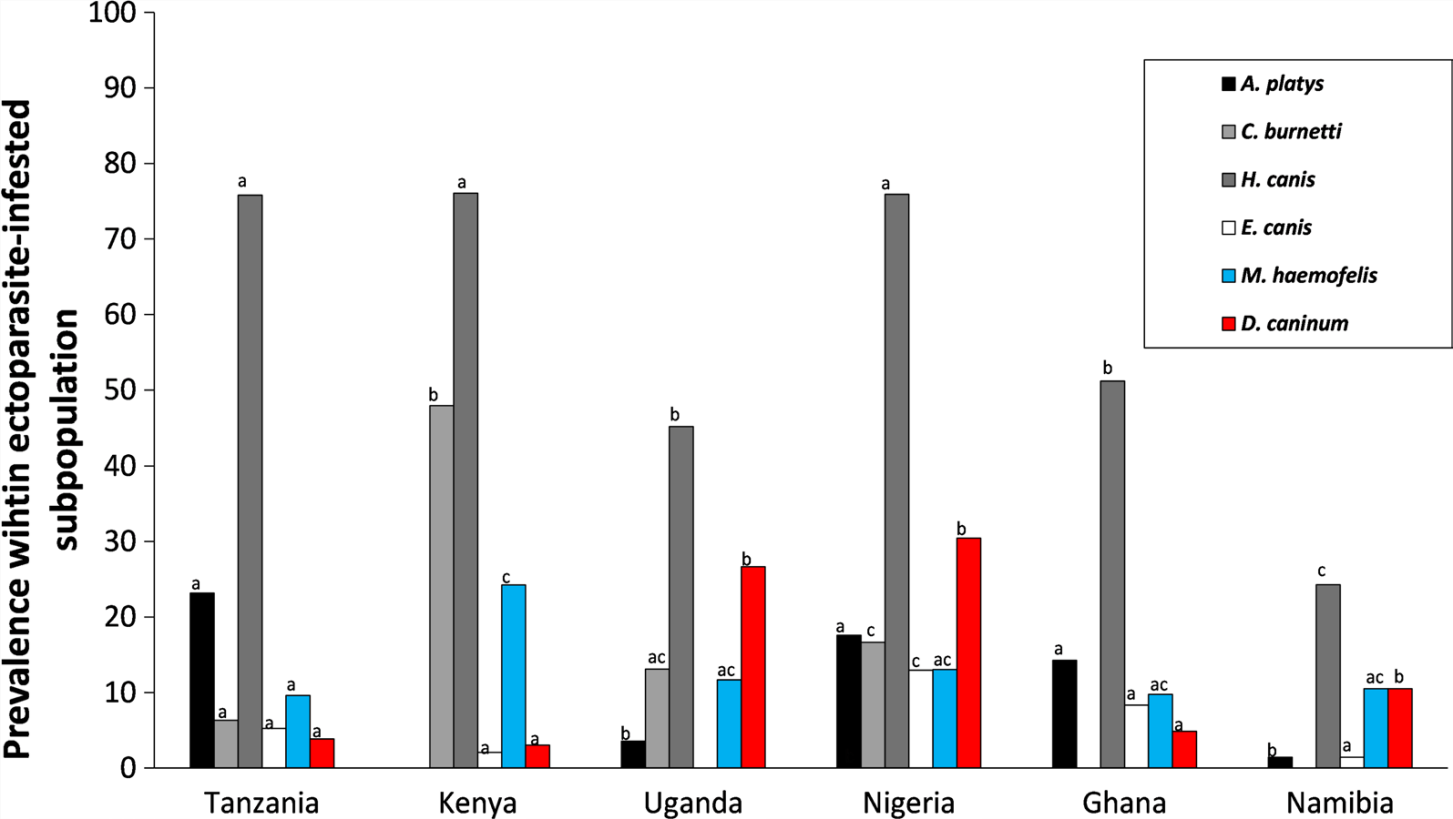
Macro-geographic variation in pathogen prevalence in ectoparasites isolated from dogs. Percentages of pools of ticks collected from dogs that were infected with one of the common tick-borne (gray shading) and flea-borne (red and blue) pathogens (overall prevalence > 5%; see Table  6). For each pathogen, the same letters above columns indicate that the the contrast between countries is not statistically different from zero

Pathogen prevalence in 261 flea pools and 537 tick pools collected from infested dog individuals in urban and rural areas of six African countries. No pair-wise comparisons have been performed for countries in which less than three dog individuals have been sampled in one of its habitats. For statistical outcomes on macrogeographic contrasts, see Fig. 5

***P < 0.001, **P < 0.01, ns (not-significant) *P* > 0.05

An overview of distributions across countries and habitat types is shown in Fig. 5 and Table 6. In the infected pools of ticks (*N* = 369), the most prevalent co-infection was *C. burnetti × H. canis* (12.2% of pools with at least one pathogen; Additional file 1: Table S5). In rural Nigeria and Kenya, > 50% of infected pools had > 1 pathogenic agent. Comparison of the pathogen distributions among countries based on PCR analyses showed that Namibia did not differ from the four other countries (pairwise comparisons with Uganda, Tanzania, Nigeria, Ghana: all *P*-values > 0.11), while all other pairwise country comparisons did (*P* < 0.047). Habitat differences (urban vs rural) within each country were found in Nigeria and Ghana, with higher pathogen diversities in their rural areas (see Table 6 for Shannon indexes).

#### Fleas

In the 261 flea pools, *M. haemofelis* (overall prevalence: 14.2%) was found in Kenya and Uganda (range: 10.0–30.8%) and in urban Tanzania, Ghana and Namibia (11.8–16%). The prevalence of *D. caninum* was > 20% in Uganda and rural Nigeria, it was regularly observed in urban Namibia (11.8%), but it was not common in other regions (< 6%). *Bartonella henselae*-infected fleas were not collected, except in the urban Ghana setting (8.3%). Co-infection (9.4%) was less common in the infected flea pools (*N* = 64) than in the tick pools, although it should be noted that the odds of detecting co-infected individuals were lower due to the low number of pathogenic agents that were screened for (Additional file 1: Table S6). Of 15 pairwise comparisons on flea-borne pathogen community distributions, 11 did show differences (all *P*-values > 0.098); significant habitat differences were not found in any country (see Table 6 for Shannon indexes).

#### Ecological correlations

In all analyses we corrected for macro-geographic spatial variation (at the country level), but allowed for habitat-related contrasts in ectoparasite loads to drive potential associations (i.e. urbanisation level was not included in the models). Prevalence of a small number of pathogens was high enough to fit reliable models (tick-borne pathogens: *A. platys*, *H. canis*, *E. canis* and *C. burnetti*; flea-borne pathogens: *M. haemofelis*, *D. caninum;* seroprevalence: *Anaplasma* spp. and *Ehrlichia* spp.) and this for a selected number of countries (i.e. countries where prevalences < 10% were excluded). Therefore, comparisons of ecological correlations between pathogens should not be over-interpreted, as different sets of countries are used for each pathogen**.** Given the high number of tests performed on the same dataset and the higher likelihood for Type I-errors, only results with *P* < 0.01 will be discussed (see "Statistical analysis").

#### Tick-borne pathogens in blood and ticks

The presence of pathogens in the ticks was explained by its presence in the dog’s blood (range Logit estimates: 1.41 ± 0.24–4.35 ± 0.33; all *P* < 0.01; Table 7). Additional variation was explained by the tick loads in *H. canis* (*R. sanguineus*: 0.33 ± 0.11; *Z* = 8.74; *P* = 0.0031) and *C. burnetti* (*H. leachi*: 0.44 ± 0.15; *Z* = 8.66; *P* = 0.0032 and *Haemaphysalis* sp.: 0.47 ± 0.15; *Z* = 9.97; *P* = 0.0016). Dogs in better body condition had less *H. canis* infected ticks (− 0.47 ± 0.17; *Z* = 7.79; *P* = 0.0053), but surprisingly this association was not found for the blood infections. Blood prevalence of *A. platys* (0.42 ± 0.12; *Z* = 13.89; *P* < 0.001) and *E. canis* (0.25 ± 0.10; *Z* = 6.26; *P* = 0.012), as well as seroprevalences for *Anaplasma* spp. (0.77 ± 0.09; *Z* = 81.10; *P* < 0.001), were positively associated with *R. sanguineus* tick loads. Furthermore, seroprevalence of *Anaplasma* spp. was positively associated with *Rhipicephalus* sp. (0.91 ± 0.16; *Z* = 33.07; *P* < 0.001) and *H. leachi* (0.42 ± 0.13; *Z* = 11.56; *P* < 0.001). *Ehrlichia* spp. seroprevalences tended to be higher in older dogs (0.011 ± 0.004; *Z* = 9.42; *P* = 0.0022; Additional file 1: Table S7). For all abovementioned pathogens found in the blood, deworming drug-treated individuals had lower prevalence than the individuals that had never been treated.

**Table 7:** Ecological models for tick-borne pathogens in host blood and ticks from infested dogs

Covariate	*A. Platys*: Ta, Na, Ni, Gh		*H. canis*: all countries		*E. canis*: Ta, Na, Ke, Ni, Gh		*C. burnetti* (tick only): Ke, Ug, Ni
	Blood	Tick	Blood	Tick	Blood	Tick	
Age (months)					− 0.38 ± 0.16*		
Body condition				− 0.47 ± 0.17**			
Tick loads							
* R. sanguineus*	0.42 ± 0.12***			0.33 ± 0.11**	0.25 ± 0.10**		
* Rhipicephalus* sp.							
* H. leachi*					− 0.48 ± 0.25*		0.44 ± 0.15**
* Haemaphysalis* spp.				0.42 ± 0.18*			0.47 ± 0.15**
Deworming^a^							
< 1 month	− 1.55 ± 0.44***		− 1.26 ± 0.31***		− 0.74 ± 0.36***		
1–6 months	− 1.58 ± 0.38***		− 1.25 ± 0.32***		− 0.36 ± 0.29***		
> 6 months	− 1.10 ± 0.43**		− 1.00 ± 0.38**		− 0.68 ± 0.40**		
Pathogen in blood tissue^b^							
Yes–No		4.00 ± 0.30***		1.41 ± 0.24***		4.35 ± 0.33***	3.02 ± 1.12**

Parameter estimates (± empirical standard error) from the logistic regressions (GEEs) that model the pathogen prevalence (levels: 0, 1) in host blood and the ticks. Only countries for which at least one area had a prevalence > 10% were included. The main assumption here is that pathogen in the blood is driven by vector presence (proxy: ticks found on dogs) and the dog’s physiology; therefore, extrinsic characteristics that correct for tick presence (urban vs rural; housing conditions; dogs around) are not included. We assume macro-geographic variation in pathogen (wildlife) reservoirs at the country level; therefore ‘country’ remained in all of the models.

Sex of dog, dogs in the environment and ectoparasiticide treatment did not significantly explain any of the variation, and therefore are not shown in the table

***P < 0.001, **P < 0.01, *P < 0.05

^a^Contrasts with group of dogs that have never been treated with a deworming drug

^b^Only included in the analyses on pathogens in feeding ticks

#### Flea-borne pathogens

Pathogen presence was not explained by any of the dog’s intrinsic and extrinsic exposure risk factors (at a significance level *α* = 0.05; see Additional file 1: Table S8 for tendencies).

#### Pathogen–tick associations

An explicit analysis of those pools of ticks in which the DNA of only a single species was detected (Table 8) again showed a higher occurrence of *C. burnetti* in the genus *Haemaphysalis* (*H. elliptica*: 55%; *Haemaphysalis* sp.: 40%) compared to the genus *Rhipicephalus* (*R. sanguineus*: 7.5%; *Rhipicephalus* sp. 10%). Also, in *R. conorii*, *Haemaphysalis* ticks (10.0–17.3%) were more often infected than* Rhipicephalus* ticks (12.7–20.0%). The opposite was true for *A. platys*, which was more often found in the genus *Rhipicephalus* (*R. sanguineus*: 12.7%; *Rhipicephalus* sp.: 20.0%) than in the genus *Haemaphysalis* (*H. elliptica*: 0.0%; *Haemaphysalis* sp.: 1.3%). *Hepatozoon canis*, on the other hand, was found in reasonable numbers (> 45%) in the most prevalent tick taxa (*R. sanguineus*, *Rhipicephalus* sp., *H. elliptica* and *Haemaphysalis* sp.). Only tick taxa with ≥ 20 individuals were considered in these enumerations.

**Table 8:** Pathogen-tick associations

	*R. sanguineus* (1)	*R. appendiculatus*	*R. simus*	*Rhipicephalus* spp. (2)	*H. elliptica* (3)	*H. spinulosa*	*Haemaphysalis* spp. (4)	*A. variegatum*	*Ixodes* spp.
*A. phagocytophilum*	0.0	0.0	0.0	0.0	0.0	0.0	0.0	0.0	0.0
*B. canis*	0.0	0.0	0.0	0.0	0.0	0.0	0.0	0.0	0.0
*B. rossi*	0.6	0.0	0.0	0.0	5.0	0.0	9.3	0.0	50.0
*D. immitis*	0.3	0.0	0.0	0.0	0.0	0.0	0.0	0.0	0.0
*E. chaffeensis*	0.0	0.0	0.0	0.0	0.0	0.0	0.0	0.0	0.0
*E. canis*	7.8	0.0	0.0	0.0	0.0	0.0	0.0	0.0	50.0
*H. canis*	63.0 a,2,3,4	100.0	80.0	46.7 c, 1	75.0 b, 1	20.0	65.3 a, 1	100.0	100.0
*A. platys*	12.7 a	0.0	0.0	20.0 a	0.0 b	0.0	1.3 b	0.0	0.0
*R. africae*	0.6	0.0	0.0	0.0	0.0	0.0	1.3	100.0	0.0
*R. conorii*	0.3 a, 3,4	0.0	0.0	3.3 a	10.0 b,1	0.0	17.3 b, 1	0.0	0.0
*C. burnetti*	7.5 a, 3,4	50.0	0.0	10.0 a	55.0 b, 1	0.0	40.0 b, 1	0.0	0.0
Tick samples	332	2	5	30	20	5	75	1	2

Only the dogs in which a single tick taxon was observed (based on the extractions of the set of pooled ticks) were included in the analysis. Statistical analyses on the occurrence of pathogens (*H. canis*, *A. platys*, *R. conorii* and *C. burnetti*) were done on tick taxa with ≥ 20 individuals (*R. sanguineus*, *Rhipicephalus* spp., *H. elliptica*, *Haemaphysalis* spp.). Africa-wide comparison: within a row, same letters behind prevalences indicate no significant difference. Country-corrected comparisons: numbers (see column headings for tick reference numbers) refer to the tick species from which the prevalence differs (P < 0.05). For this latter analysis, in the following groups of countries a sufficient number of dogs was sampled to allow for pairwise statistical comparisons. Tanzania, Namibia, Uganda: *Rhipicephalus* spp. vs *R. sanguineus*; Ghana, Kenya, Uganda: *Haemaphysalis* spp. vs *R. sanguineus*; Kenya, Uganda: *H. elliptica* vs (*R. sanguineus* and *Haemaphysalis* spp.); Uganda: *R. sanguineus *vs (*Rhipicephalus* spp., *Haemaphysalis* spp., *H. elliptica*)

Africa-wide comparison: same lowercase letters indicate no significant difference. Country-corrected comparisons: for pathogens in each tick species’ column, pathogens followed by a number is linked with a significant difference (*P* < 0.05) with one of the other tick species: (*R. sanguineus *(1),* Rhipicephalus* spp. (2),* H. elliptica* (3),* Haemaphysalis* sp. (4)

For the following groups of countries a sufficient number of dogs were sampled to allow for pairwise statistical comparisons between tick taxa: Tanzania, Namibia, Uganda: *Rhipicephalus* sp. vs *R. sanguineus*; Ghana, Kenya, Uganda: *Haemaphysalis* spp. vs *R. sanguineus*; Kenya, Uganda: *H. elliptica* vs (*R. sanguineus* and *Haemaphysalis* sp.); Uganda: *R. sanguineus* vs (*Rhipicephalus* spp., *Haemaphysalis* sp., *H. elliptica*)

## Discussion

The objective of the study was to determine the most important vector-borne pathogens and ectoparasites of dogs in six sub-Saharan African countries. We handled the data collection using a rigorous pre-defined protocol and did a meta-analysis on standardized data. The occurrence of ticks, fleas and pathogens in both vector and host were investigated, focusing on strong contrasts between broad country-specific urban categories (see "Methods" section). Several surveys on ticks and their pathogens have been conducted in Africa, but these mainly consider production animals in agricultural areas [13]. This study shows that, despite the significant socio-economic value of companion animals to humans, they represent an indirect risk for zoonotic vector-borne diseases in a One Health perspective by hosting pathogenic agents and their vectors. As dogs are in close contact to wildlife and to production animals (e.g. cattle), they are at the interface of several vertebrate communities, although with different functionalities to humans.

More than 70% of the dogs sampled were infected with at least one vector-borne pathogen in the blood, and infection rates were even higher in the vectors collected from these infected dogs. Even in the absence of any thorough knowledge on local host abundances and diversity, several of the associations and geographical patterns turn out to be the consequence of the tick vectors’ biology. *Rhipicephalus sanguineus*, commonly known as the brown dog tick [14], is found worldwide in warmer climates and is a monotropic (dogs) three-host tick. It is an endophilic tick that completes its entire life-cycle indoors, such as inside kennels. It typically shows negative geotropism after feeding, a behavior in which the ticks climb to the walls of borrows or man-made shelters and hide in cracks and crevices to oviposit or molt to the next stage. Therefore, it is not surprising that in most countries (with the exception of Namibia, where sampling happened during the dry season) the tick was more often found in urban areas than rural areas, and found more often on dogs that were restricted to their yards (i.e. closer to outdoor man-made structures, like kennels). However, not all *Rhipicephalus* ticks are endophilic, which is likely the reason why the group of unidentified ticks belonging to this genus has a less outspoken preference for rural or urban areas (Table 3). Adults of *R. appendiculatus*,* R. microplus*, *R. simus* and *R. senegalensis* are all linked to cattle and wildlife such as buffalo and large antelope. Furthermore, with the exception of *R. microplus*, all of these ticks are considered to be three-host ticks with di- or telotropic behavior, making their life-cycle and preferences far more complicated than those of *R. sanguineus* [15]. In our extensive survey, few dogs were found to be infested with *R. microplus*.

In comparison, most *Haemaphysalis* spp. ticks are from wildlife and considered to be exophilic [16]. As a consequence, several identified (*H. leachi*, *H. elliptica*,* H. spinulosa*) and undefined *Haemaphysalis* ticks were observed more often on dogs from rural areas than on those from urban areas. In *H. leachi* and *H. elliptica* (previously considered as *H. leachi* as well [17]), adults parasitize domestic and wild carnivores, while the immature stages feed on rodents. For those countries where there were sufficient data to allow statistical analysis on the latter species (Kenya and Uganda), free-roaming dogs—often going into wildlife habitats—indeed did show higher prevalences. *Haemaphysalis spinulosa* adults appear to feed on various small- and medium-sized carnivores, as well as hedgehogs [16]. A very high proportion of individuals belonging to the genus *Haemaphysalis* could not be identified, but genetic clustering revealed a large group taxon that most likely involves a new or genetically uncharacterized species (manuscript in preparation).

Twelve vector-borne pathogens were detected in host tissue and vectors. For the most common tick-borne pathogens, we found a strong correlation between their presence in host blood and their presence in the ticks, without any indication of a causative correlation or vector competence. When the distributions of the pathogens with respect to tick species are considered, the most striking contrast is the high prevalence of *C. burnetti* in the genus *Haemaphysalis* in which all members of the genus are known to be vector-competent for this pathogen [18]. Surprisingly, only few dog blood samples tested positive for this pathogen despite individual dogs carrying infected ticks. Ticks could have been infected in the nymphal stage, when feeding on reservoir competent hosts. The pathogen was also not observed in ticks from Ghana and Namibia. The occurrence of *H. canis* was higher when dogs were more heavily infested (with both *Rhipicephalus* spp. and *Haemaphysalis* spp.), but the pathogen did not show a strong preference for a particular tick species, indicating that both genera could equally contribute to *H. canis*’ transmission—via ingestion of infected ticks. *Ehrlichia canis* and *A. platys* prevalences in the blood were positively correlated with *R. sanguineus*, as were the *Anaplasma* sp. and—to a lower extent— *Ehrlichia* sp. seroprevalences, indicating the central role of *R. sanguineus* ticks in pathogen exposure in domestic dogs across almost the complete African continent. *Rickettsia conorii* was absent in host blood and mainly found in the East African countries. We emphasize that prevalences (both in ectoparasites and vector-borne pathogens) are likely affected by parasite-induced host mortality, which especially in highly virulent vector-borne pathogens (e.g. *B. rossi* often found in the genus *Haemaphysalis* [19, 20]) could strongly influence our interpretation of the presented cross-sectional data. *Borrelia* seroprevalence was close to zero, likely because of the low prevalence of *Ixodes* species, the main vectors of *Borrelia burgdorferi* sensu lato [21].

By far the most abundant flea species found on dogs was *C. felis*, carrying *M. haemofelis* and *D. caninum*. Interestingly, associations with deworming drugs were found for fleas and ticks. Dogs that were recently treated carried the most ticks, whereas the longer in the past the treatment (or no treatment at all), the lower the tick counts. For fleas and *Haemaphysalis* sp. we observed patterns in the opposite direction: untreated dogs had overall more ectoparasites than treated individuals. Although these trends are contradictory, we could hypothesize that dog owners would more likely treat their dog when ectoparasites are observed. Knowing fleas and ticks belonging to the genus *Haemaphysalis* are more difficult to observe visually because of their small size, the likelihood of a dog being treated might directly be related to the size of the ectoparasites.

Additional screening for mosquito-, tsetse- and sand fly-borne pathogens resulted in a low number of observed cases. *Dirofilaria immitis* seroprevalence was very low, despite its much higher prevalence in dog blood. The discrepancy between antigen level and PCR results may be explained in a number of ways. First, the molecular assay employed in this study was able to detect samples with very low levels of microfilaremia that could not be detected by the less sensitive serological test. Positive PCR signals, indicative of *D. immitis* DNA, could have amplified DNA from other filarioids commonly found in dogs. A subset of the presumed *D. immitis*-positive DNA samples was post-hoc subjected to sequencing, which revealed *Acanthocheilonema reconditum* and *Dirofilaria repens* (using BLAST and NCBI database). Secondly, blocked antigen may be a consequence of the presence of immune complexes, which lowers sensitivity of the antigen detection. Protocols such as heat treatment might disrupt these complexes and allow detection of the antigen. Also, if a dog has an infection that was established < 4 months previously, immature *D. immitis* may be present (identified by PCR) but the antigen test will be negative regardless of how it is performed. Finally, it has been shown that in dogs infected with only one or two adult females, or only male worms, antigen is unlikely going to be detected [22].

A few additional considerations should be noted regarding the outcomes of this study. Many of the correlations may be the results of macro-geographic and habitat differences, which come with differences in vector, host and pathogen communities. Explanatory analyses of correlative data at this large scale are very often affected by biases and confounders (e.g. effects of wildlife population density) that cannot be controlled for due to a lack of information. Furthermore, as the main objective was to determine the most important ectoparasites and vector-borne pathogens, the focus of this study was on the subpopulation of infested individuals, which is why outcomes have to be interpreted in terms of occurrence and diversity, rather than true prevalence. With regard to the vector-borne pathogens, we consider bias should be less strong, as pathogen occurrence in the body is the outcome of past infestations (which not necessarily correlates with the infestation levels at capture). But here also, since tissue tropism may heavily differ among pathogens and show (unknown) temporal patterns herein, blood screening does not allow every pathogen to be identified with equal probability. Nevertheless, to the best of our knowledge, this work forms the most extensive and standardized study in sub-Saharan countries so far, giving an overview of important vectors and vector-borne pathogens; as such, the information could serve as baseline data for future research and interventions.

We also found tick species and pathogens that are not classically associated with companion animals but which still have the potential to transmit zoonotic disease-causing pathogens in dogs. High levels of co-infestation and co-infections were observed, adding to the zoonotic risk, given the high potential of bridging opportunities to cattle and humans via vectors and/or immuno-modulations and atypical virulence patterns (due to co-parasitism). Furthermore, we found multi-host ticks in urban areas, which have the potential to extend the network of pathogen transmission to humans.

## Conclusions

This standardized surveillance underscores the importance of ectoparasites and their pathogens in dogs of sub-Saharan Africa, with co-parasitism being the rule rather than the exception. Future research needs to include wildlife host surveys, tick densities in the off-host environment, detailed habitat characteristics and specific resources that may support dense populations of wildlife hosts. Furthermore, species-specific responses to space characteristics [23] in least-cost path analyses that make use of habitat connectivity will substantially increase our understanding of how spatial elements could affect local vector-borne pathogen risk. Integration of this knowledge with a good understanding of current complexities in socio-economic and climate changes will enable policymakers and scientists to develop and provide prevention strategies.

## Supplementary Information

**Additional file 1: Fig. S1.** Overview of sampling times and average seasonal variation in precipitation and temperature. **Table S1.** Distribution of PCR signals allocated to an ectoparasite taxon (identification at genus level and more precise) in the infested dogs of urban and rural areas. **Table S2.** Distribution of co-infested dogs within the subpopulation of tick-infested dogs. **Table S3.** Co-infestations by different flea species (identification at genus level and lower). **Table S4.** Co-infections in dog blood. **Table S5.** Co-infections in dog ticks. **Table S6.** Co-infections in dog fleas. **Table S7.** Correlations with sero-prevalences. **Table S8.** Correlations with flea-borne pathogens.

**Additional file 2**. Capture form.

## Data Availability

The datasets used and/or analyzed during the current study are available from the corresponding author on reasonable request.

## Abbreviations

BLAST: Basic local alignment search tool
DNA: Deoxyribonucleic acid
GEE: Generalized estimation equation (model)
mtDNA: Mitochondrial DNA
qPCR: Quantitative real-time polymerase chain reaction
