# Supplementary material for: A community approach of pathogens and their arthropod vectors (ticks and fleas) in dogs of African Sub-Sahara
Source: Parasit Vectors. 2021 Nov 16;14:576. doi: 10.1186/s13071-021-05014-8 (PMC8594167; doi:10.1186/s13071-021-05014-8)
Supplement: Supplementary file 1 — Additional file 1: Fig. S1. Overview of sampling times and average seasonal variation in precipitation and temperature. Table S1. Distribution of PCR signals allocated to an ectoparasite taxon (identification at genus level and more precise) in the infested dogs of urban and rural areas. Table S2. Distribution of co-infested dogs within the subpopulation of tick-infested dogs. Table S3. Co-infestations by different flea species (identification at genus level and lower). Table S4. Co-infections in dog blood. Table S5. Co-infections in dog ticks. Table S6. Co-infections in dog fleas. Table S7. Correlations with sero-prevalences. Table S8. Correlations with flea-borne pathogens. [file 13071_2021_5014_MOESM1_ESM.docx]

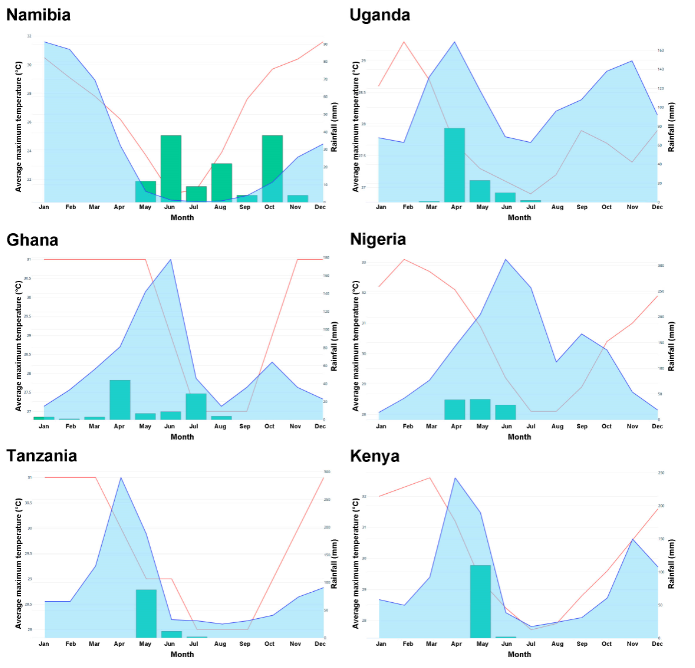


**Fig. S1:** Overview of the moments of sampling within the average seasonal variation in precipitation and temperature (data obtained from https://weather-and-climate.com). Except for Namibia, the majority of samples were retrieved during the rainy season.

**Table S1.** Distribution of PCR-signals allocated to an ectoparasite taxon (identification at genus level and more precise) in the infested dogs of urban and rural areas in six African countries (Percentages sum to 100% for each country x habitat combination).

|  |  |  | Tanzania (%) | | Kenya (%) | | Uganda (%) | | Nigeria (%) | | Ghana (%) | | Namibia (%) | |
| --- | --- | --- | --- | --- | --- | --- | --- | --- | --- | --- | --- | --- | --- | --- |
|  | Species | Overall | Rural | Urban | Rural | Urban | Rural | Urban | Rural | Urban | Rural | Urban | Rural | Urban |
| Ticks | *R. sanguineus* | 64.48 | 66.67 | 87.76 | **24.71** | **66.67** | **2.63** | **46.34** | 79.41 | 90.74 | **64.29** | **97.67** | 90.32 | 76.92 |
|  | *R. appendiculatus* | 0.36 | 0.00 | 0.00 | 0.00 | 0.00 | 2.63 | 2.44 | 0.00 | 0.00 | 0.00 | 0.00 | 0.00 | 0.00 |
|  | *R. simus* | 1.95 | 0.00 | 2.04 | 8.24 | 0.00 | 0.00 | 0.00 | 1.47 | 0.00 | 0.00 | 0.00 | 0.00 | 5.13 |
|  | *R. microplus* | 0.18 | 2.08 | 0.00 | 0.00 | 0.00 | 0.00 | 0.00 | 0.00 | 0.00 | 0.00 | 0.00 | 0.00 | 0.00 |
|  | *R. senegalensis* | 0.18 | 0.00 | 0.00 | 0.00 | 0.00 | 0.00 | 0.00 | 0.00 | 1.85 | 0.00 | 0.00 | 0.00 | 0.00 |
|  | *Rhipicephalus* sp. | 6.39 | **25.00** | **6.12** | 0.00 | 0.00 | 2.63 | 17.07 | 2.94 | 0.00 | 2.38 | 0.00 | 9.68 | 17.95 |
|  | *H. elliptica* | 6.22 | 0.00 | 0.00 | 16.47 | 4.17 | 13.16 | 9.76 | 7.35 | 7.41 | 4.76 | 0.00 | 0.00 | 0.00 |
|  | *H. leachi* | 0.53 | 0.00 | 0.00 | 0.00 | 0.00 | 0.00 | 0.00 | 4.41 | 0.00 | 0.00 | 0.00 | 0.00 | 0.00 |
|  | *H. spinulosa* | 1.42 | 2.08 | 2.04 | 0.00 | 0.00 | 13.16 | 2.44 | 0.00 | 0.00 | 0.00 | 0.00 | 0.00 | 0.00 |
|  | *Haemaphysalis* sp. | 16.52 | 0.00 | 0.00 | **50.59** | **29.17** | **63.16** | **21.95** | 2.94 | 0.00 | **16.67** | **2.33** | 0.00 | 0.00 |
|  | *A. variegatum* | 0.36 | 0.00 | 0.00 | 0.00 | 0.00 | 0.00 | 0.00 | 1.47 | 0.00 | 2.38 | 0.00 | 0.00 | 0.00 |
|  | *Amblyomma* sp. | 0.89 | 2.08 | 0.00 | 0.00 | 0.00 | 2.63 | 0.00 | 0.00 | 0.00 | 7.14 | 0.00 | 0.00 | 0.00 |
|  | *Ixodes* sp. | 0.53 | 2.08 | 2.04 | 0.00 | 0.00 | 0.00 | 0.00 | 0.00 | 0.00 | 2.38 | 0.00 | 0.00 | 0.00 |
|  | Tick-infested dogs | 563 | 48 | 49 | 85 | 24 | 38 | 41 | 68 | 54 | 42 | 43 | 31 | 39 |
|  |  |  |  |  |  |  |  |  |  |  |  |  |  |  |
| Fleas | *C. felis* | 93.31 | 100.00 | 96.30 | 89.66 | 100.00 | 100.00 | 100.00 | 100.00 | 100.00 | 84.21 | 95.65 | 100.00 | 58.82 |
|  | *E. gallinacea* | 0.37 | 0.00 | 0.00 | 0.00 | 0.00 | 0.00 | 0.00 | 0.00 | 0.00 | 5.26 | 0.00 | 0.00 | 0.00 |
|  | *Echidnophaga* sp. | 6.32 | 0.00 | 3.70 | 10.34 | 0.00 | 0.00 | 0.00 | 0.00 | 0.00 | 10.53 | 4.35 | 0.00 | 41.18 |
|  | Flea-infested dogs | 269 | 27 | 27 | 58 | 13 | 40 | 20 | 21 | 1 | 19 | 23 | 2 | 17 |
|  |  |  |  |  |  |  |  |  |  |  |  |  |  |  |
|  | | | | | | | | | | | | | | |

Note: Per dog, an extraction on a pooled set of ticks and fleas was done, before the PCR-analysis was executed; maximum one PCR-positive per dog could be obtained for each of the taxa investigated.

In bold prevalence for tick batches ≥ 5 individuals

**Table S2.** Distribution of co-infested dogs within the subpopulation of tick-infested dogs.

|  |  | Tanzania (%) | | Kenya (%) | | Uganda (%) | | Nigeria (%) | | Ghana (%) | | Namibia (%) | |
| --- | --- | --- | --- | --- | --- | --- | --- | --- | --- | --- | --- | --- | --- |
| Species and co-infestions | Overall | Rural | Urban | Rural | Urban | Rural | Urban | Rural | Urban | Rural | Urban | Rural | Urban |
| *R. sanguineus* | 63.88 | 68.89 | 87.23 | 16.67 | 65.22 | 2.78 | 44.74 | 77.19 | 92.16 | 66.67 | 97.67 | 90.32 | 75.00 |
| *Haemaphysalis* sp. | 14.56 | 0.00 | 0.00 | 48.61 | 26.09 | 61.11 | 15.79 | 1.75 | 0.00 | 11.11 | 2.33 | 0.00 | 0.00 |
| *Rhipicephalus* sp. | 5.83 | 24.44 | 6.38 | 0.00 | 0.00 | 2.78 | 15.79 | 0.00 | 0.00 | 2.78 | 0.00 | 9.68 | 13.89 |
| *H. elliptica* | 3.88 | 0.00 | 0.00 | 12.50 | 4.35 | 13.89 | 10.53 | 0.00 | 1.96 | 0.00 | 0.00 | 0.00 | 0.00 |
| *H. elliptica x R. sanguineus* | 1.94 | 0.00 | 0.00 | 2.78 | 0.00 | 0.00 | 0.00 | 8.77 | 3.92 | 2.78 | 0.00 | 0.00 | 0.00 |
| *Haemaphysalis* sp. *x R. sanguineus* | 1.94 | 0.00 | 0.00 | 8.33 | 4.35 | 0.00 | 5.26 | 0.00 | 0.00 | 2.78 | 0.00 | 0.00 | 0.00 |
| *H. spinulosa* | 0.97 | 0.00 | 0.00 | 0.00 | 0.00 | 11.11 | 2.63 | 0.00 | 0.00 | 0.00 | 0.00 | 0.00 | 0.00 |
| *R. simus* | 0.97 | 0.00 | 0.00 | 5.56 | 0.00 | 0.00 | 0.00 | 0.00 | 0.00 | 0.00 | 0.00 | 0.00 | 2.78 |
| *R. sanguineus x Rhipicephalus* sp. | 0.78 | 0.00 | 0.00 | 0.00 | 0.00 | 0.00 | 0.00 | 3.51 | 0.00 | 0.00 | 0.00 | 0.00 | 5.56 |
| *Amblyomma* sp. *x Haemaphysalis* sp. | 0.58 | 0.00 | 0.00 | 0.00 | 0.00 | 2.78 | 0.00 | 0.00 | 0.00 | 5.56 | 0.00 | 0.00 | 0.00 |
| *R. sanguineus x R. simus* | 0.58 | 0.00 | 2.13 | 0.00 | 0.00 | 0.00 | 0.00 | 1.75 | 0.00 | 0.00 | 0.00 | 0.00 | 2.78 |
| *H. leachi x R. sanguineus* | 0.39 | 0.00 | 0.00 | 0.00 | 0.00 | 0.00 | 0.00 | 3.51 | 0.00 | 0.00 | 0.00 | 0.00 | 0.00 |
| *Ixodes* sp. | 0.39 | 0.00 | 2.13 | 0.00 | 0.00 | 0.00 | 0.00 | 0.00 | 0.00 | 2.78 | 0.00 | 0.00 | 0.00 |
| *R. appendiculatus* | 0.39 | 0.00 | 0.00 | 0.00 | 0.00 | 2.78 | 2.63 | 0.00 | 0.00 | 0.00 | 0.00 | 0.00 | 0.00 |
| *A. variegatum* | 0.19 | 0.00 | 0.00 | 0.00 | 0.00 | 0.00 | 0.00 | 1.75 | 0.00 | 0.00 | 0.00 | 0.00 | 0.00 |
| *A. variegatum x R. sanguineus* | 0.19 | 0.00 | 0.00 | 0.00 | 0.00 | 0.00 | 0.00 | 0.00 | 0.00 | 2.78 | 0.00 | 0.00 | 0.00 |
| *Amblyomma* sp. *x H. elliptica* | 0.19 | 0.00 | 0.00 | 0.00 | 0.00 | 0.00 | 0.00 | 0.00 | 0.00 | 2.78 | 0.00 | 0.00 | 0.00 |
| *Amblyomma* sp. *x R. sanguineus* | 0.19 | 2.22 | 0.00 | 0.00 | 0.00 | 0.00 | 0.00 | 0.00 | 0.00 | 0.00 | 0.00 | 0.00 | 0.00 |
| *H. elliptica x Haemaphysalis* sp. | 0.19 | 0.00 | 0.00 | 1.39 | 0.00 | 0.00 | 0.00 | 0.00 | 0.00 | 0.00 | 0.00 | 0.00 | 0.00 |
| *H. elliptica x R. sanguineus x R. simu* | 0.19 | 0.00 | 0.00 | 1.39 | 0.00 | 0.00 | 0.00 | 0.00 | 0.00 | 0.00 | 0.00 | 0.00 | 0.00 |
| *H. elliptica x R. senegalensis* | 0.19 | 0.00 | 0.00 | 0.00 | 0.00 | 0.00 | 0.00 | 0.00 | 1.96 | 0.00 | 0.00 | 0.00 | 0.00 |
| *H. elliptica x R. simus* | 0.19 | 0.00 | 0.00 | 1.39 | 0.00 | 0.00 | 0.00 | 0.00 | 0.00 | 0.00 | 0.00 | 0.00 | 0.00 |
| *H. leachi x Haemaphysalis* sp. | 0.19 | 0.00 | 0.00 | 0.00 | 0.00 | 0.00 | 0.00 | 1.75 | 0.00 | 0.00 | 0.00 | 0.00 | 0.00 |
| *H. spinulosa x Haemaphysalis* sp. | 0.19 | 0.00 | 0.00 | 0.00 | 0.00 | 2.78 | 0.00 | 0.00 | 0.00 | 0.00 | 0.00 | 0.00 | 0.00 |
| *H. spinulosa x Ixodes* sp. | 0.19 | 2.22 | 0.00 | 0.00 | 0.00 | 0.00 | 0.00 | 0.00 | 0.00 | 0.00 | 0.00 | 0.00 | 0.00 |
| *H. spinulosa x R. sanguineus* | 0.19 | 0.00 | 2.13 | 0.00 | 0.00 | 0.00 | 0.00 | 0.00 | 0.00 | 0.00 | 0.00 | 0.00 | 0.00 |
| *Haemaphysalis* sp. *x R. simus* | 0.19 | 0.00 | 0.00 | 1.39 | 0.00 | 0.00 | 0.00 | 0.00 | 0.00 | 0.00 | 0.00 | 0.00 | 0.00 |
| *Haemaphysalis* sp. *x Rhipicephalus* sp. | 0.19 | 0.00 | 0.00 | 0.00 | 0.00 | 0.00 | 2.63 | 0.00 | 0.00 | 0.00 | 0.00 | 0.00 | 0.00 |
| *R. microplus x Rhipicephalus* sp. | 0.19 | 2.22 | 0.00 | 0.00 | 0.00 | 0.00 | 0.00 | 0.00 | 0.00 | 0.00 | 0.00 | 0.00 | 0.00 |
| Co-infested dogs (%) | 8.93 | 6.67 | 4.26 | 16.67 | 4.35 | 5.56 | 7.89 | 19.30 | 5.88 | 16.67 | 0.00 | 0.00 | 8.33 |
| N° of infested dogs | 515 | 45 | 47 | 72 | 23 | 36 | 38 | 57 | 51 | 36 | 43 | 31 | 36 |
|  | | | | | | | | | | | | | |

Note: Percentages refer to dogs with one or more tick species feeding on them. From top to bottom: highest to lowest combinations observed. identification at genus level and lower. (Percentages sum to 100% for each country x habitat combination).

**Table S3.** Co-infestations by different flea species (identification at genus level and lower).

|  |  | Tanzania (%) | | Kenya (%) | | Uganda (%) | | Nigeria (%) | | Ghana (%) | | Namibia (%) | |
| --- | --- | --- | --- | --- | --- | --- | --- | --- | --- | --- | --- | --- | --- |
| Flea species and co-infestions | Overall | Rural | Urban | Rural | Urban | Rural | Urban | Rural | Urban | Rural | Urban | Rural | Urban |
| *C. felis* | 93.05 | 100.00 | 96.15 | 88.68 | 100.00 | 100.00 | 100.00 | 100.00 | 100.00 | 82.35 | 95.65 | 100.00 | 56.25 |
| *Echidnophaga* sp. | 3.47 | 0.00 | 0.00 | 1.89 | 0.00 | 0.00 | 0.00 | 0.00 | 0.00 | 5.88 | 4.35 | 0.00 | 37.50 |
| *C. felis x Echidnophaga* sp. | 3.09 | 0.00 | 3.85 | 9.43 | 0.00 | 0.00 | 0.00 | 0.00 | 0.00 | 5.88 | 0.00 | 0.00 | 6.25 |
| *C. felis x E. gallinacea* | 0.39 | 0.00 | 0.00 | 0.00 | 0.00 | 0.00 | 0.00 | 0.00 | 0.00 | 5.88 | 0.00 | 0.00 | 0.00 |
| Co-infested individuals (%) | 3.48 | 0.00 | 3.85 | 9.43 | 0.00 | 0.00 | 0.00 | 0.00 | 0.00 | 11.76 | 0.00 | 0.00 | 6.25 |
| N° of infested dogs | 259 | 27 | 26 | 53 | 13 | 40 | 20 | 21 | 1 | 17 | 23 | 2 | 16 |
|  | | | | | | | | | | | | | |

Note: Prevalences refer to the number of host individuals with one or more flea species feeding on them. From top to bottom: highest to lowest combinations observed.

**Table S4.** Co-infections in dog blood.

|  |  | Tanzania (%) | | Kenya (%) | | Uganda (%) | | Nigeria (%) | | Ghana (%) | | Namibia (%) | |
| --- | --- | --- | --- | --- | --- | --- | --- | --- | --- | --- | --- | --- | --- |
| Species and co-infestions | Overall | Rural | Urban | Rural | Urban | Rural | Urban | Rural | Urban | Rural | Urban | Rural | Urban |
| *H. canis* | 54.84 | 53.66 | 55.00 | 70.59 | 60.00 | 91.84 | 81.82 | 31.82 | 38.10 | 44.12 | 17.95 | 36.84 | 25.00 |
| *H. canis x E. canis* | 10.14 | 17.07 | 7.50 | 10.29 | 33.33 | 0.00 | 4.55 | 13.64 | 14.29 | 11.76 | 15.38 | 0.00 | 5.00 |
| *E. canis* | 6.91 | 9.76 | 2.50 | 1.47 | 0.00 | 0.00 | 0.00 | 4.55 | 19.05 | 2.94 | 5.13 | 31.58 | 45.00 |
| *A. platys x H. canis* | 5.99 | 7.32 | 12.50 | 1.47 | 0.00 | 0.00 | 6.82 | 9.09 | 0.00 | 14.71 | 7.69 | 10.53 | 0.00 |
| *A. platys* | 5.3 | 4.88 | 0.00 | 0.00 | 0.00 | 0.00 | 0.00 | 11.36 | 14.29 | 5.88 | 15.38 | 15.79 | 10.00 |
| *A. platys x E. canis* | 3.69 | 2.44 | 5.00 | 0.00 | 0.00 | 0.00 | 0.00 | 9.09 | 4.76 | 8.82 | 2.56 | 5.26 | 15.00 |
| *A. platys x H. canis x E. canis* | 3.23 | 0.00 | 7.50 | 0.00 | 0.00 | 0.00 | 0.00 | 11.36 | 0.00 | 2.94 | 12.82 | 0.00 | 0.00 |
| *B. rossi x H. canis* | 3 | 0.00 | 0.00 | 5.88 | 0.00 | 8.16 | 4.55 | 2.27 | 9.52 | 0.00 | 0.00 | 0.00 | 0.00 |
| *H. canis x D. immitis* | 1.61 | 4.88 | 2.50 | 0.00 | 0.00 | 0.00 | 0.00 | 0.00 | 0.00 | 2.94 | 7.69 | 0.00 | 0.00 |
| *B. rossi* | 1.38 | 0.00 | 2.50 | 2.94 | 6.67 | 0.00 | 2.27 | 0.00 | 0.00 | 0.00 | 2.56 | 0.00 | 0.00 |
| *D. immitis* | 0.69 | 0.00 | 2.50 | 0.00 | 0.00 | 0.00 | 0.00 | 0.00 | 0.00 | 2.94 | 2.56 | 0.00 | 0.00 |
| *A. platys x H. canis x D. immitis* | 0.46 | 0.00 | 0.00 | 0.00 | 0.00 | 0.00 | 0.00 | 0.00 | 0.00 | 0.00 | 5.13 | 0.00 | 0.00 |
| *C. burnetti x H. canis* | 0.46 | 0.00 | 0.00 | 1.47 | 0.00 | 0.00 | 0.00 | 2.27 | 0.00 | 0.00 | 0.00 | 0.00 | 0.00 |
| *D. immitis x E. canis* | 0.46 | 0.00 | 0.00 | 0.00 | 0.00 | 0.00 | 0.00 | 0.00 | 0.00 | 0.00 | 5.13 | 0.00 | 0.00 |
| *A. platys x C. burnetti x H. canis x E. canis* | 0.23 | 0.00 | 0.00 | 1.47 | 0.00 | 0.00 | 0.00 | 0.00 | 0.00 | 0.00 | 0.00 | 0.00 | 0.00 |
| *A. platys x H. canis x D. immitis x E. canis* | 0.23 | 0.00 | 2.50 | 0.00 | 0.00 | 0.00 | 0.00 | 0.00 | 0.00 | 0.00 | 0.00 | 0.00 | 0.00 |
| *A. platys x H. canis x Trypanosoma* spp. | 0.23 | 0.00 | 0.00 | 0.00 | 0.00 | 0.00 | 0.00 | 2.27 | 0.00 | 0.00 | 0.00 | 0.00 | 0.00 |
| *B. rossi x A. platys x H. canis* | 0.23 | 0.00 | 0.00 | 1.47 | 0.00 | 0.00 | 0.00 | 0.00 | 0.00 | 0.00 | 0.00 | 0.00 | 0.00 |
| *B. rossi x D. immitis* | 0.23 | 0.00 | 0.00 | 1.47 | 0.00 | 0.00 | 0.00 | 0.00 | 0.00 | 0.00 | 0.00 | 0.00 | 0.00 |
| *B. rossi x E. canis* | 0.23 | 0.00 | 0.00 | 0.00 | 0.00 | 0.00 | 0.00 | 2.27 | 0.00 | 0.00 | 0.00 | 0.00 | 0.00 |
| *B. rossi x H. canis x E. canis* | 0.23 | 0.00 | 0.00 | 1.47 | 0.00 | 0.00 | 0.00 | 0.00 | 0.00 | 0.00 | 0.00 | 0.00 | 0.00 |
| *H. canis x D. immitis x E. canis* | 0.23 | 0.00 | 0.00 | 0.00 | 0.00 | 0.00 | 0.00 | 0.00 | 0.00 | 2.94 | 0.00 | 0.00 | 0.00 |
|  |  |  |  |  |  |  |  |  |  |  |  |  |  |
| Co-infected individuals (%) | 30.87 | 31.71 | 37.5 | 25.00 | 33.33 | 8.16 | 15.91 | 52.27 | 28.57 | 44.12 | 56.41 | 15.79 | 20.00 |
| N° of infested dogs with at least one pathogen | 434 | 41 | 40 | 68 | 15 | 49 | 44 | 44 | 21 | 34 | 39 | 19 | 20 |
|  | | | | | | | | | | | | | |

Note: Prevalences refer to the number of host individuals with one or more pathogens. From top to bottom: highest to lowest prevalence observed.

**Table S5.** Co-infections in dog ticks.

|  |  | Tanzania (%) | | Kenya (%) | | Uganda (%) | | Nigeria (%) | | Ghana (%) | | Namibia (%) | |
| --- | --- | --- | --- | --- | --- | --- | --- | --- | --- | --- | --- | --- | --- |
| (co-)infections | Overall | Rural | Urban | Rural | Urban | Rural | Urban | Rural | Urban | Rural | Urban | Rural | Urban |
| *H. canis* | 52.03 | 52.63 | 61.54 | 25.00 | 100.00 | 56.52 | 57.14 | 36.84 | 66.67 | 50.00 | 66.67 | 88.89 | 77.78 |
| *C. burnetti x H. canis* | 12.2 | 7.89 | 2.56 | 35.29 | 0.00 | 21.74 | 3.57 | 19.30 | 0.00 | 0.00 | 0.00 | 0.00 | 0.00 |
| *A. platys x H. canis* | 7.59 | 10.53 | 23.08 | 0.00 | 0.00 | 0.00 | 3.57 | 12.28 | 6.06 | 11.54 | 4.17 | 0.00 | 11.11 |
| *H. canis x E. canis* | 3.52 | 2.63 | 2.56 | 1.47 | 0.00 | 0.00 | 0.00 | 7.02 | 12.12 | 3.85 | 0.00 | 11.11 | 0.00 |
| *A. platys* | 3.25 | 7.89 | 2.56 | 0.00 | 0.00 | 0.00 | 7.14 | 5.26 | 0.00 | 3.85 | 8.33 | 0.00 | 0.00 |
| *C. burnetti* | 2.98 | 0.00 | 0.00 | 10.29 | 0.00 | 0.00 | 10.71 | 1.75 | 0.00 | 0.00 | 0.00 | 0.00 | 0.00 |
| *A. platys x H. canis x E. canis* | 2.71 | 0.00 | 7.69 | 0.00 | 0.00 | 0.00 | 0.00 | 3.51 | 0.00 | 7.69 | 12.50 | 0.00 | 0.00 |
| *R. conorii x C. burnetti x H. canis* | 2.71 | 0.00 | 0.00 | 13.24 | 0.00 | 4.35 | 0.00 | 0.00 | 0.00 | 0.00 | 0.00 | 0.00 | 0.00 |
| *A. platys x C. burnetti x H. canis* | 1.63 | 5.26 | 0.00 | 0.00 | 0.00 | 0.00 | 0.00 | 7.02 | 0.00 | 0.00 | 0.00 | 0.00 | 0.00 |
| *R. africae* | 1.63 | 2.63 | 0.00 | 0.00 | 0.00 | 13.04 | 0.00 | 0.00 | 0.00 | 3.85 | 4.17 | 0.00 | 0.00 |
| *B. rossi* | 1.36 | 0.00 | 0.00 | 1.47 | 0.00 | 0.00 | 7.14 | 1.75 | 0.00 | 3.85 | 0.00 | 0.00 | 0.00 |
| *B. rossi x C. burnetti x H. canis* | 1.08 | 0.00 | 0.00 | 4.41 | 0.00 | 0.00 | 0.00 | 0.00 | 3.03 | 0.00 | 0.00 | 0.00 | 0.00 |
| *E. canis* | 1.08 | 0.00 | 0.00 | 0.00 | 0.00 | 0.00 | 0.00 | 0.00 | 9.09 | 3.85 | 0.00 | 0.00 | 0.00 |
| *R. africae x H. canis* | 1.08 | 2.63 | 0.00 | 0.00 | 0.00 | 0.00 | 0.00 | 1.75 | 0.00 | 7.69 | 0.00 | 0.00 | 0.00 |
| *R. conorii* | 1.08 | 0.00 | 0.00 | 1.47 | 0.00 | 4.35 | 3.57 | 0.00 | 0.00 | 0.00 | 0.00 | 0.00 | 11.11 |
| *B. felis x H. canis* | 0.81 | 5.26 | 0.00 | 0.00 | 0.00 | 0.00 | 0.00 | 1.75 | 0.00 | 0.00 | 0.00 | 0.00 | 0.00 |
| *R. conorii x H. canis* | 0.81 | 2.63 | 0.00 | 2.94 | 0.00 | 0.00 | 0.00 | 0.00 | 0.00 | 0.00 | 0.00 | 0.00 | 0.00 |
| *B. rossi x H. canis* | 0.54 | 0.00 | 0.00 | 0.00 | 0.00 | 0.00 | 0.00 | 0.00 | 3.03 | 3.85 | 0.00 | 0.00 | 0.00 |
| *A. platys x C. burnetti x H. canis x E. canis* | 0.27 | 0.00 | 0.00 | 0.00 | 0.00 | 0.00 | 0.00 | 1.75 | 0.00 | 0.00 | 0.00 | 0.00 | 0.00 |
| *B. rossi x C. burnetti* | 0.27 | 0.00 | 0.00 | 1.47 | 0.00 | 0.00 | 0.00 | 0.00 | 0.00 | 0.00 | 0.00 | 0.00 | 0.00 |
| *B. rossi x R. conorii x H. canis* | 0.27 | 0.00 | 0.00 | 0.00 | 0.00 | 0.00 | 3.57 | 0.00 | 0.00 | 0.00 | 0.00 | 0.00 | 0.00 |
| *H. canis x D. immitis* | 0.27 | 0.00 | 0.00 | 0.00 | 0.00 | 0.00 | 0.00 | 0.00 | 0.00 | 0.00 | 4.17 | 0.00 | 0.00 |
| *R. africae x C. burnetti x H. canis* | 0.27 | 0.00 | 0.00 | 1.47 | 0.00 | 0.00 | 0.00 | 0.00 | 0.00 | 0.00 | 0.00 | 0.00 | 0.00 |
| *R. conorii x C. burnetti* | 0.27 | 0.00 | 0.00 | 0.00 | 0.00 | 0.00 | 3.57 | 0.00 | 0.00 | 0.00 | 0.00 | 0.00 | 0.00 |
| Co-infected individuals (%) | 36.59 | 36.84 | 35.9 | 61.76 | 0 | 26.09 | 14.29 | 54.39 | 24.24 | 34.62 | 20.83 | 11.11 | 11.11 |
| N° of infested dogs with at least one pathogen found in the tick pool | 369 | 38 | 39 | 68 | 15 | 23 | 28 | 57 | 33 | 26 | 24 | 9 | 9 |
|  | | | | | | | | | | | | | |

Note: Prevalences refer to tick pools (given extractions at dog individual level have been obtained of a pool of ticks) with one or more pathogen species. From top to bottom: highest to lowest prevalences observed.

**Table S6.** Co-infections in dog fleas.

|  |  | Tanzania (%) | | Kenya (%) | | Uganda (%) | | Nigeria (%) | | Ghana (%) | | Namibia (%) | |
| --- | --- | --- | --- | --- | --- | --- | --- | --- | --- | --- | --- | --- | --- |
| (co-)infections | Overall | Rural | Urban | Rural | Urban | Rural | Urban | Rural | Urban | Rural | Urban | Rural | Urban |
| *M. haemofelis* | 48.44 | 50.00 | 80.00 | 84.62 | 100.00 | 26.67 | 16.67 | 0.00 | 0.00 | 50.00 | 50.00 | 0.00 | 50.00 |
| *D. caninum* | 39.06 | 50.00 | 20.00 | 7.69 | 0.00 | 66.67 | 66.67 | 57.14 | 0.00 | 50.00 | 16.67 | 0.00 | 50.00 |
| *M. haemofelis x D. caninum* | 9.38 | 0.00 | 0.00 | 7.69 | 0.00 | 6.67 | 16.67 | 42.86 | 0.00 | 0.00 | 0.00 | 0.00 | 0.00 |
| *B. henselae* | 3.13 | 0.00 | 0.00 | 0.00 | 0.00 | 0.00 | 0.00 | 0.00 | 0.00 | 0.00 | 33.33 | 0.00 | 0.00 |
| Co-infected fleas (%) | 9.38 | 0.00 | 0.00 | 7.69 | 0 | 6.67 | 16.67 | 42.86 | 0.00 | 0.00 | 0.00 | 0.00 | 0.00 |
| N° of infested dogs with at least one pathogen found in the flea pool | 64 | 2 | 5 | 13 | 4 | 15 | 6 | 7 | 0 | 2 | 6 | 0 | 4 |
|  | | | | | | | | | | | | | |

Note: Prevalences refer to flea pools (given extractions at dog individual level have been obtained of a pool of fleas) with one or more pathogens.

**Table S7.** Correlations with sero-prevalences.

|  | *Anaplasma*  *Ta, Ke, Ug, Ni, Na* | *Ehrlichia*  *All countries* |
| --- | --- | --- |
| Covariate |  |  |
|  |  |  |
| Age (months) |  | 0.011+0.004** |
|  |  |  |
| Tick species loads |  |  |
| *R_sanguineus* | 0.77 + 0.09*** | 0.24+0.12* |
| *Rhipicephalus* sp. | 0.91 + 0.16*** |  |
|  |  |  |
| *H. leachi* | 0.45 + 0.13** |  |
| *Haemaphysalis* sp. |  | 0.40+0.20* |
|  |  |  |
| Pathogen in blood tissue ^$^ |  |  |
| Yes-No | ^£^ | 0.73+0.26** ^$^ |
|  | | |

Note: Parameter estimates (+ empirical standard error) from the logistic regressions (GEE’s) that model the seroprevalence ~~prevalence~~ (levels: 0, 1). Only countries for which at least one area had a prevalence of at least 10 % were included. Country differences have been omitted from the table, but were included in all analyses.

Ta(nzania), Ug(anda), Na(mibia), Ke(nya), Ni(geria), Gh(ana)

P < 0.001: ***; < 0.01: **; P < 0.05: *, _ P > 0.05

£ *A. platys*; $ *E. canis*

For both antibody-responses, the following factors did not significantly explain any of the pathogen variation (P > 0.05) and were excluded from the model and table: sex, body condition, dogs in environment and parasite treatments

**Table S8.** Correlations with flea-borne pathogens.

|  | *M. haemofelis*  *(fleas only)* | *D. caninum*  *(fleas only)*  *Ug, Ni, Na* |
| --- | --- | --- |
| Covariate |  |  |
|  |  |  |
| Sex |  |  |
| Female - Male |  | 1.08 + 0.55 * |
|  |  |  |
| # Dogs around | -0.24 + 0.12* | 0.26 + 0.12* |
|  | | |

Note: Parameter estimates (+ empirical standard error) from the logit-regressions (GEE’s) that model the flea-borne pathogen prevalence (levels: 0, 1) in the dogs’ blood. Only countries with an overall prevalence of at least 10 % were included. Country differences have been omitted from the table, but were included in all analyses. Ug(anda), Na(mibia),Ni(geria)

P < 0.001: ***; < 0.01: **; P < 0.05: *, P > 0.05

Housing, age, body condition, deworming and *C. felis* loads did not explain pathogen variation (P > 0.05), and therefore were excluded from the model
