# Supplementary material for: A community approach of pathogens and their arthropod vectors (ticks and fleas) in dogs of African Sub-Sahara
Source: Parasit Vectors. 2021 Nov 16;14:576. doi: 10.1186/s13071-021-05014-8 (PMC8594167; doi:10.1186/s13071-021-05014-8)
Supplement: Supplementary file 2 — Additional file 2. Capture form. [file 13071_2021_5014_MOESM2_ESM.pdf]

**DCF 1**	**FIELD ASSESSMENT (Individual animal)**	(Fix barcode in this box)

Time = 24 hour clock Date format dd/mmm/yyyy

**Date**		**Time**		**Animal no.**	

Owner consent, animal details and history: O Dog O Cat to participate in survey

**Owner :** I give mermission for my animal to participate (as per information sheet):	**Signature:**		**Date:**
**Age (months)**		**Breed**	
**Sex**	O Male O Female		
**Diet**	O Homemade O Tinned O Kibble O Mixed		
**Last deworming:**	O Never O 1-6 months ago O < 1 month ago O >6 months ago	**Dewormer used:**	
**Last ectoparasiticide**	O Never O 1-6 months ago O < 1 month ago O >6 months ago	**Ectoparasiticide used:**	
**Last vaccination**	O Never O Within last 3 years O Within last year	**Vaccines used:**	☐ Rabies ☐ Leptospira ☐ DHP ☐ CPi/*Bordetella* ☐ Other: ____________
**Housing**	O Indoor O Free roaming O Yard	**Other pets?**	O Yes O No
**If "yes" to "Other pets": Cats or dogs and how many?**		O Dog(s) ____________ O Cat(s)____________	

Geographical details

**Pet origin (name of city/town/village)**		**GPS coordinates of sampling site**	

Physical examination and general health assessment

**Any major abnormal clinical signs evident?**	O Yes O No	**If yes, provide details below:**	
**Body Condition Score**	O 1 O 2 O 3 O 4 O 5	**Mucous membrane colour**	O Normal (pink & moist) O Pale O Icteric O Other ____________
**Temperature (°C)**		**Pulse (beats/minute)**	
**Respiration rate (/minute)**			

Ectoparasite assessments

**Ticks**	**Area 1**	O 1 ticks O 2 ticks O ≥ 3 ticks
	**Area 2**	O 1 ticks O 2 ticks O ≥ 3 ticks
	**Area 3**	O 1 ticks O 2 ticks O ≥ 3 ticks
	**Area 4**	O 1 ticks O 2 ticks O ≥ 3 ticks
	**Area 5**	O 1 ticks O 2 ticks O ≥ 3 ticks
	**Area 6**	O 1 ticks O 2 ticks O ≥ 3 ticks
	**Area 7**	O 1 ticks O 2 ticks O ≥ 3 ticks
**Fleas**	**Overall**	O 1-10 fleas O 11-50 fleas O >50 fleas
**Lice**	**Overall**	O 1-10 lice O 11-50 lice O >50 lice

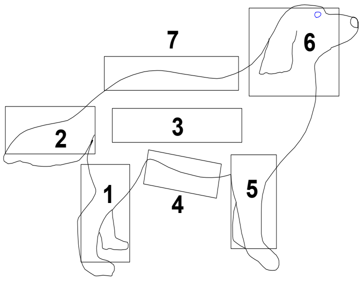

1. Outside hind legs
2. Tail and anal area
3. Lateral area, not shoulders
4. Abdomen & inside hind legs
5. Shoulder and fore-legs
6. Head and neck
7. Dorsal, shoulders to tail base

Ectoparasite specimen collection (single jar with 70% ethanol per animal)

**Up to 30 ticks collected:**	O Yes O No	**Fleas and lice:**	O Collected as many as possible O None present
**Ensured that the barcode on the collection jar corresponds with barcode on this form**	O Yes		

Blood collection and processing and anal swab preparation

**Blood specimen collected**	O Yes O No	**FTA card used**	O Yes O No	**4DxPlus kit used**	O Yes O No
**4Dx Plus kit positive results:**	☐ Heartworm ☐ *Borrelia burgdorferi* (Lyme disease) ☐ *Anaplasma* spp. ☐ *Ehrlichia* spp.				
**Serum prepared**	O Yes O No	**Anal swab performed**	O Yes O No		
**Rabies vaccine administered?**	O Yes O No	**Ectoparasite preventive dispensed?**	O Yes O No		
**Field assessment performed by:**	**Signature**		**Date:**		

Preparation for shipment checklist

**Following was prepared for shipment to Clinvet:**	☐ DCF 1 ☐ FTA card (one half) ☐ Ectoparasite collection jar ☐ Anal swab		
**Prepared for shipment by:**	**Signature**		**Date:**
